# Multiscale Process Modelling in Translational Systems Biology of *Leishmania major*: A Holistic view

**DOI:** 10.1038/s41598-020-57640-4

**Published:** 2020-01-21

**Authors:** Nutan Chauhan, Shailza Singh

**Affiliations:** National Centre for Cell Science, NCCS Complex, Ganeshkhind, SP Pune University Campus, Pune, 411007 India

**Keywords:** Parasitic infection, Stochastic networks

## Abstract

Present work aims to utilize systems biology and molecular modelling approach to understand the inhibition kinetics of *Leishmania major* GLO I and identifying potential hit followed by their validation through *in vitro* and animal studies. Simulation of GLO I inhibition has shown to affect reaction fluxes of almost all reactions in the model that led to increased production of various AGEs and free radicals. Further, *in vitro* testing of C1 and C2, selected through molecular modelling revealed remarkable morphological alterations like size reduction, membrane blebbing and loss in motility of the parasite, however, only C1 showed better antileishmanial activity. Additionally, C1 showed apoptosis mediated leishmanicidal activity (apoptosis-like cell death) along with cell-cycle arrest at sub-G0/G1 phase and exhibited potent anti-leishmanial effect against intracellular amastigotes. Furthermore, decrease in parasite load was also observed in C1 treated BALB/c female mice. Our results indicate that C1 has healing effect in infected mice and effectively reduced the parasitic burden. Hence, we suggest C1 as a lead molecule which on further modification, may be used to develop novel therapeutics against *Leishmaniasis*.

## Introduction

Pathogenic parasites trypanosomatids belong to the class Kinetoplastida and have been extensively studied for a long time due to their association with many neglected tropical diseases^1,2^ in humans as well as in animals and plants. They are predominantly possessing a monoxenous life-cycle (restricted to only invertebrates), yet, several of them have dixenous life-cycle in which their transmission to vertebrates is facilitated by a sandfly vector. Among these parasites, many species of *Leishmania* causes different clinical forms of leishmaniasis known as cutaneous leishmaniasis (CL), mucocutaneous leishmaniasis (MCL), and the most life threatening one, visceral leishmania (VL). CL is the most common clinical forms of leishmaniasis and is caused by various species of *Leishmania* including *Leishmania major*. This disease has always been neglected as a major public health problem due to its non-fatality, however, major consequences of the disease consists of skin lesions leading to scars. Current chemotherapies are inadequate and suffer by various problems like toxicity, side effects and emerging drug resistance. These issues have raised concerns among researchers for developing more effective and affordable drugs as well as potential therapeutic molecular drug targets.

Recent studies show that instead of focusing on single enzyme and/or protein, researchers nowadays are putting their efforts in analysing metabolic networks to identify potential drug targets in many organisms^3–6^. Kinetic modeling, genome scale metabolic modeling, and constraint-based methods etc. are such techniques that have been extensively used in many studies for finding the importance of enzymes in various pathways including redox metabolism^7–13^. Among these techniques, kinetic modelling requires experimental kinetic parameters and knowledge of rate laws. There are several studies in which kinetic modelling has been used to study the kinetics of gene transfer and antibiotic resistance in several bacteria^14,15^.

In trypanosomatids, a unique redox metabolism exists that help the parasites to deal with the oxidative stresses that can be host originated (inside parasite infected macrophages) or parasite originated. In both the cases, the stress is posed mainly by free radicals generated via many pathways. Additionally, several reactive metabolites, *viz*., glyoxals, apart from their nature to modify macromolecules, can also play important role in free radicals formation. Methylglyoxal (MGO), a dicarbonyl glyoxal, is one of the highly reactive metabolites having affinity towards amino groups of proteins and nucleic acids. It is a spontaneous product of glycolysis pathway and formed from dihydroxyacetone (DHAP) and/or glyceraldehyde-3-phosphate (GA3P)^16,17^. MGO is found to irreversibly modify ~0.1–2% arginine residues of proteins and one in ten bases of DNA thereby causing their inactivation and leading to the formation of advanced glycation end products (AGEs)^18–25^. Therefore, MGO needs to be neutralized before producing any harm to the cells. The most important pathway for its detoxification is glyoxalase pathway that consists of two enzymes, glyoxalase I (GLO I) and glyoxalase II (GLO II)^26,27^. GLO I, first, isomerizes hemithioacetal (HTA), a spontaneous product formed after MGO-T[SH]_2_ conjugation, to S-D-lactoyaltrypanothione followed by GLO II mediated conversion to D-lactate. In this pathway, GLO I mediated step is described as rate limiting and the enzyme is found to have essential role in the survival of *Leishmania*^28^. In our previous work, we also identify GLO I as an important drug target through systems biology approach^29^. Hence, in current work, we seek to understand the kinetics of GLO I through *in silico* kinetic modeling followed by the identification of potential hit compounds by molecular modeling and dynamics simulation approaches. The identified potential hits were further tested experimentally to check their *in vitro* and *in vivo* potency against *Leishmania major*.

## Materials and Method

### Kinetic modelling and sensitivity analysis

Construction of all kinetic models was done using CellDesigner v4.4^30^. After drawing, these models were saved as Systems Biology Markup Language (SMBL) file and imported in COPASI (COmplex Pathway Simulator) v4.19.140^31^ followed by assignment of appropriate kinetic laws. All reactions were considered as irreversible for maintaining uniformity in the kinetic model (Table 1). The concentrations, time units and reaction fluxes in the model are in molar (M), seconds and M/s, respectively. All the kinetic parameters were obtained/calculated from published literature (Supplementary File 1, Table 2, S1). To observe the effect of different parameters on the models, parameter scanning and sensitivity analyses were performed using deterministic LSODA ODE (ordinary differential equations) solver^32^.

**Table 1:** Basic properties of kinetic models.

Model Number	Reactions^#^	Kinetic Parameters^#^	Components^#^	Kinetic laws^#^
M1a	4	6	6	4
M1b	4	7	6	4
M2a	39	70	52	39
M2b	39	71	52	39

^#^**Reactions**: Total number of Reactions in a particular model system; **Kinetic Parameters**: Total kinetic parameters (for example: Km, Vm, etc) used in all reactions in a particular model system; **Components**: Total Components (metabolites/protein) in a particular model; **Kinetic Laws**: Total number of kinetic laws (kinetic mechanism) involved in a particular model system.

### Molecular modelling and pharmacophore model development

Benzimidazoles and thiosemicarbazones derivatives were retrieved from various online databases (PubChem, ZINC, DrugBank) and prepared by applying OPLS2005 force field followed by their ADME/Tox screening in QikProp module (Schrodinger, LLC). Receptor preparation and docking was done in protein Preparation Wizard module and Glide, respectively, with default parameters.

MD simulation using Desmond (v 4.4) was performed on selected complexes solvated in an orthorhombic box filled with SPC water molecules and neutralized by adding Na^+^ ions at random places. The entire system was minimized using steepest decent algorithm until a gradient threshold of 25 Kcal/mol/Å was reached. The Martyna-Tobias-Klein scheme was used for pressure coupling. Electrostatic forces were calculated using the PME algorithm^33^. All runs were performed at 300 K at constant volume and temperature (NPT ensemble) under certain periodic boundary conditions (explicit solvent simulations with periodic boundary conditions using orthorhombic box distance (Å) 10.0, 10.0, 10.0; Na^+^ ions: 15) for a period of 10 ns –100 ns with the time steps of 2 fs.

Pharmacophore hypotheses describing the important inherent chemical features responsible for the biological activity were generated using Discovery Studio software.

### *In vitro* culture of parasites and culture condition

Wild type *Leishmania major* promastigotes (MHOM/IL/67/JERICHOII) were grown in RPMI-1640 media supplemented with 20% heat inactivated fetal bovine serum (FBS) (GIBCOBRL, Grand Island, NY) and antibiotics at 27 °C. For all experiments, parasite cultures in logarithmic (exponential) growth phase were considered.

### Analysis of drug sensitivity by colorimetric MTT assay

#### Anti-leishmanial activity assay on L. major promastigotes by colorimetric MTT assay

The compounds were dissolved in DMSO to prepare a stock of 100 mM and further diluted in R20 (RPMI with 20% (v/v) heat inactivated FBS) to make fresh working solution of 1 mM concentration. The final concentration of DMSO was kept below 0.4% (v/v). Exponential growth phase promastigotes were resuspended in fresh medium to achieve 1 × 10^6^ cells/ml and seeded in 96-well culture plates in triplicates. The concentration of compounds was used ranging from 1 to 1000 μM. The plates were incubated at 27 °C for 48 h followed by PBS wash. The viability of the parasites was evaluated by using quantitative colorimetric MTT (3-[4,5-methylthiazol-2-yl]-2,5-diphenyltetrazoliumbromide) assay to assess the metabolic activity of the cells. Briefly, 20 μl of MTT labelling reagent (5 mg/ml) and 180 μl PBS was added to each well including the controls (non-treated) and incubated in dark for a time period of 4 h at 27 °C. As an indicator of cell viability, the formazan crystals, were dissolved in 100 μl of DMSO per well to obtain a blue solution. The absorbance was read spectrophotometrically at 570 nm. A decrease in the amount of MTT converted indicates toxicity to the cell. Tetrazolium dye reduction is generally assumed to be dependent of NAD(P)H dependent cellular oxidoreductase enzymes present largely in cytosolic compartment of viable cells. Therefore, the reduction of MTT depends on the cellular metabolic activity due to NAD(P)H flux. These enzymes reduce the tetrazolium dye (MTT) to its insoluble formazan purple coloured crystals inside the cells. Cells with low metabolism (or dead cells) reduce very little or negligible MTT in comparison to rapidly dividing cells^34^. Hence, in our work, cells treated with C1 showed less MTT reduction thereby less formazan formation and low spectrophotometric absorbance demonstrating cytotoxicity of C1 to the parasite. The IC50 (drug concentrations at which 50% of parasites were inhibited) was calculated by regression analysis. The results are expressed as the means and standard deviations of three independent experiments.

#### Macrophage infections and drug susceptibility assay against intracellular amastigote forms

Raw cell line macrophages were seeded (1 × 10^5^ cells/ml) onto coverslips and incubated at 37 °C for 12 h. After washing with PBS (pH 7), cells were infected with stationary phase *L. major* promastigotes (1 × 10^6^ cells/ml) at a macrophage to parasite ratio of 1:10 and incubated at 37 °C for 24 h for proper internalization of parasite. Further, the cells were washed with PBS and given appropriate treatments with C1 in all groups except control and incubated for at 37 °C. After 48 h incubation, cells were given PBS wash followed by DAPI staining. Amastigote IC50 was calculated by computing parasite infectivity index in untreated and treated groups by counting the number of amastigotes inside the infected macrophage (amastigotes/macrophage)^35^.

#### Mammalian cell cytotoxicity

The cytotoxicity assay for compounds was performed on macrophage cell line (Raw 264.7 cell line). The macrophage cells were seeded in 96 well plates (1 × 10^5^ cells/ml) in DMEM media (GIBCO BRL, Grand Island, NY) supplemented with 10% heat inactivated FBS and 100 μg/ml penicillin-streptomycin. Cells were incubated at 37 °C, 5% CO_2_ for a period of 24 h. This was followed by the media replacement with fresh medium containing the compound (80–200 µM), and the cells again were incubated for 48 h at same culture condition. The assay was performed as mentioned above and %Cell viability in relation to untreated controls was calculated

### Detection of phosphatidylserine exposure

For detecting phosphatidylserine exposure, double staining with FITC Annexin V apoptosis detection kit with PI (Propidium Iodide), Biolegend, was used. Briefly, the untreated and treated promastigotes (1 × 10^6^ cells/ml) were incubated at 27 °C for 48 h followed by PBS washing and centrifuged at 14,00 g for 10 min. The pellets were then resuspended in the standard provided with the kit followed by double staining with annexin V-FITC and PI as per the manufacturer’s instructions. The parasites were incubated in dark for 15–20 min at 26 °C before recording the intensity of labelled parasites by capturing 10,000 events on FACS Calibur flow cytometer (Becton Dickinson). For analysis, CellQuest software was used and the percentage of positive cells was plotted and assessed in the form of respective dot plots and histograms.

### DNA content analysis for cell cycle

The untreated and treated promastigotes (1 × 10^6^ cells/ml) were incubated at 27 °C for 48 h and washed with PBS and centrifuged at 14,00 g for 10 min. Pelleted cells were dissolved and fixed in 70% cold ethanol and incubated overnight at −20 °C. The fixed cells were again washed and resuspended in PBS and stained with PI (1 mg/ml). The cells were incubated for 20 min in dark at room temperature and the intensity of PI was analysed with FACS Calibur (Becton Dickinson) flow cytometer and CellQuest software by acquiring 10,000 cells.

### Morphological analysis

Briefly, 1 × 10^6^ *L. major* promastigotes were cultured with or without respective drugs at their IC50 concentrations and incubated for 48 h at 27 °C. Further, parasites were washed with PBS and smeared onto the poly L-lysine coated coverslips. Air dried cells were fixed in 2.5% glutaraldehyde solution by keeping the cells for a minimum time period of 3 h. The fixed cells were then washed thrice in PBS and dehydrated in acetone gradient (30%, 50%, 70%, 90%, and 100%; each for 5 min). Finally, cells were air dried, coated with platinum using Q150T Turbo-Pumped Sputter Coater and observed under Nova NanoSEM field emission scanning electron microscope operating at 15 kV.

### Cloning, expression and purification of *L. major* GLO I

The construct containing the *L. major* GLO I gene (437 bp) was procured from Invitrogen, Thermofisher Scientific. The synthesised gene was inserted into pET28a(+)_AF090 vector. The construct was reconstituted and confirmation of GLO I insertion was done using restriction enzymes NotI and NdeI. Competent *Escherichia coli* BL21 (DE3) cells were prepared and the construct was transformed at 42 °C for 30 sec by heat shock treatment. These cells were grown on kanamycin (50 µg/ml) containing Luria Berttini (LB) agar medium plates followed by overnight incubation at 37 °C. Kanamycin resistant single colony of BL21 (GLO I + ve) was inoculated in LB broth with kanamycin and incubated at 37 °C for 2–3 h or until an absorbance of 0.6 at 600 nm was reached. The GLO I expression was induced by 1 mM Isopropyl β-D-1-thiogalactopyranoside (IPTG) and again incubated at 27 °C for 4 h. The expression of GLO I was checked on 12% SDS-PAGE^36^.

Cell were centrifuged at 4,696 g for 30 min and lysed by resuspending the pellet in Ni-NTA lysis buffer (50 mM NaH_2_PO_4_, 300 mM NaCl, 10 mM imidazole pH 8.0) with lysozyme and incubated for 30 min on ice followed by sonication (four 30 sec on/off cycle). Further, the homogenate was centrifuged and the supernatant was poured into Ni-NTA column. The column was washed twice with Ni-NTA wash buffer (50 mM NaH2PO4, 300 mM NaCl, 20 mM imidazole, pH 8.0). GLO I was eluted with elution buffer (50 mM NaH2PO4, 300 mM NaCl, 250 mm imidazole, pH 8.0) in fractions. The purity of GLO I was checked on 12% SDS-PAGE using standard protocol.

### *In vivo* screening

Animal study was conducted according to the protocols approved by the Institutional Animal Ethical Committee of National Centre for Cell Science (NCCS, Registration number: 7/GO/ReBi/S/99/CPCSEA; Date of registration: 09/03/1999) under the Project number: EAF/2017/B-312. BALB/c female mice, 8 week old with 20–25 mg body weight were used. For infection, stationary phase *L*.*major* promastigotes (2 × 10^7^ cells/ml) were injected subcutaneously in the left hind limb footpad of the mice. Compound C1 was initially prepared in DMSO followed by its dilution in Milli-Q water. Mice were divided in groups (5 mice per group) and administered orally for 10 days as follows: control group with Milli-Q water; C1 groups with four different doses of C1 (5, 10, 20 and 40 mg/kg/body weight/per day). Mice were euthanized 12 days post treatment and treatment potency was determined by estimating parasite load in draining lymph nodes by limiting dilution assay as per the Taswell’s method^37^.

The statistical significance of differences in parasite load amongst groups was evaluated using one-way ANOVA with Tukey’s correction.

### Glyoxalase I assay

The activity of LmGLO I was assayed spectrophotometrically at room temperature. For this, the initial rate of formation of S-D-lactoyl trypanothione was measured at 240 nm as described previously^38^ with slight modifications. Briefly, DTT (3 mM) was used to reduce trypanothione disulfide (1 mM) (BOC Sciences) at 60 °C for 20 min. For preparing the assay mixture, 200 mM methylglyoxal (Sigma), 300 mM reduced trypanothione and 20 mM NiCl_2_ were added to 100 mM MOPS buffer to reach the final volume of 500 µl^39^. The assay mixture was then incubated for 10 min followed by the addition of LmGLO I (100 nM) and C1 inhibitor in various concentrations ranging from 0–320 µM for calculating the IC50. All the assays were performed in triplicate.

### Ethics statement

All mice were maintained in compliance with the Institutional Animal Ethics guidelines. All animal experiments were approved by the Institutional Animal Ethics Committee (IAEC) (7/GO/c/99/CPCSEA) of the National Centre for Cell Science (IAEC Project Number-EAF /2017/B-312).

## Results and Discussion

### Models reconstruction and kinetic simulation

**Figure 1:**
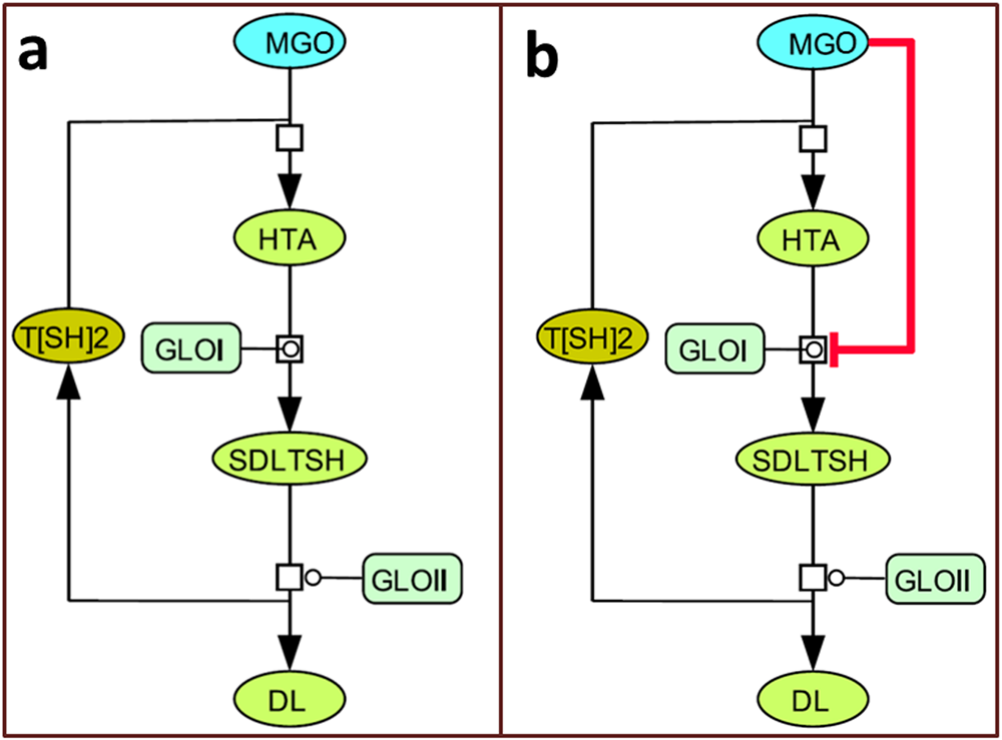
Two basic models constructed to study the inhibition kinetics of GLO I. **(a)** *M1a* (NI: noninhibited), and **(b**) *M1b* (I: inhibited) models.

Initially, two basic models for glyoxalase pathway were constructed. In wild type model (noninhibited), *M1a*, the reactions for synthesis and degradation of MGO were included. Hence, for constructing the perturbed model (inhibited), *M1b*, MGO mediated substrate level inhibition reaction for GLO I^24,25^ was introduced in *M1a* and assigned with appropriate rate law (Tables 1, 2, Table S1, Fig. 1a,b). For both models previously determined rate constants (except hypothetical Ki for GLO I) were used. The kinetics of degradation of MGO in both models was observed through time course simulation in COPASI.

**Table 2:** Different rate laws assigned to various reactions in extended kinetic model.

SN.	Reaction name	Reaction	Kinetic Law
1.	SAMsyn	Met ->SAM	Hill Cooperativity
2.	SAMdc	SAM ->dcSAM	Henri-Michaelis-Menten (irr)
3.	Arginase	Arg ->Orn	Henri-Michaelis-Menten (irr)
4.	ODC	Orn ->Put + CO_2_	Henri-Michaelis-Menten (irr)
5.	SpdS	Put + dcSAM ->Spd	Henri-Michaelis-Menten (irr)
6.	yECS	Glu + Cys ->GluCys	Henri-Michaelis-Menten (irr)
7.	GS	GluCys + Gly ->GSH	Henri-Michaelis-Menten (irr)
8.	TryS1	Spd + GSH ->Gspd	Henri-Michaelis-Menten (irr)
9.	TryS2	Gspd + GSH ->T[SH]_2_	Henri-Michaelis-Menten (irr)
10.	MGO to HTA	MGO + T[SH]_2_ ->HTA	Mass action (irr)
11.	TR	TS2 + NADPH ->T[SH]_2_ + NADP	Henri-Michaelis-Menten (irr)
12.	HTA to MGO	HTA ->MGO + T[SH]_2_	Mass action (irr)
13.	GLOI	HTA ->SDLTSH	Henri-Michaelis-Menten (irr)
14.	GLOI^#^	HTA ->SDLTSH; MGO	Substrate inhibition (irr)
15.	GLOII	SDLTSH ->DL + T[SH]_2_	Henri-Michaelis-Menten (irr)
16.	Arg modification	Arg + MGO = Arg-MGO + MGO^●−^	Mass action (irr)
17.	Lys modification	Lys + MGO = Lys-MGO + MGO^●−^	Mass action (irr)
18.	TXNo Reduction	T[SH]_2_ + TXNo ->TXNr + TS_2_	Mass action (irr)
19.	TDPx	H_2_O_2_ + TXNr ->TXNo + H_2_O	Bi Bi PingPong
20.	TryP	H_2_O_2_ + TXNr ->TXNo + H_2_O	Bi Bi PingPong
21.	Fenton reaction_ H_2_O_2_	H_2_O_2_ + Fe^3+^ ->^●^OH + Fe^2+^	Mass action (irr)
22.	O2^●−^ dismutation	2 * O_2_^●−^ + H + ->H_2_O_2_ + O_2_	Mass action (irr)
23.	SOD	2 * O_2_^●–^>H_2_O_2_ + O_2_	Mass action (irr)
24.	Lipid radical formation	^●^OH + LH ->L^●^ + H_2_O	Mass action (irr)
25.	Lipid peroxide radical formation	L^●^ + O_2_ ->LO_2_^●^	Mass action (irr)
26.	LOOH formation	LO_2_^●^ + LH ->LOOH + L^●^	Mass action (irr)
27.	Fenton reaction for Lipid	LOOH + Fe^3+^ ->LO_2_^●^ + Fe^2+^	Mass action (irr)
28.	Nonradical formation	2 * LO_2_^●^ ->MGO	Mass action (irr)
29.	NOS	Arg + NADPH ->NO^●^ + Citrulline + NADP	Henri-Michaelis-Menten (irr)
30.	NO2. formation	NO^●^ + O ->NO_2_^●^	Mass action (irr)
31.	ONOOH formation	NO_2_^●^ + ^●^OH ->ONOOH	Mass action (irr)
32.	NO2. from ONOOH	ONOOH + H2O2 ->O_2_^●−^ + NO_2_^●^ + H_2_O + H^+^	Mass action (irr)
33.	ONOO- formation	NO_2_^●^ + O_2_^●–^>ONOO^-^	Mass action (irr)
34.	TDPx reduction	TXNr + TDPxo ->TXNo + TDPxr	Mass action (irr)
35.	TDPx for NO_2_−	TDPxr + ONOO–>TDPxo + NO_2_^−^	Mass action (irr)
36.	TXN for NO_2_−	TXNr + ONOO–>TXNo + NO_2_^−^	Mass action (irr)
37.	T[SH]_2_ for NO_2_−	T[SH]_2_ + ONOO–>TS_2_ + NO_2_^−^	Mass action (irr)
38.	Lipid radical from NO_2_^−^	NO_2_^●^ + LH ->NO_2_^−^ + L^●^ + H_+_	Mass action (irr)
39.	O_2_^●−^ formation from MGO radical	MGO^●–^>MGO + O_2_^●−^	Mass action (irr)

^**#**^Perturbation reaction introduced as GLOI inhibition in Inhibited model *M1b*.

(***Abbr****:* ^*●*^*OH: Hydroxyl free radical; AGEs: Advanced Glycation End Products; Arg: Arginine; Cys: Cysteine; dcSAM: S-adenosyl methionine amine; DL: D-lactate; GLOI: GlyoxalaseI; GLOII: glyoxalaseII; Glu: Glutamate; GluCys: L-glutamyl-L-cysteine; Gly: Glycine; GS: Glutathione synthetase; GSH: Glutathione; Gspd: Glutathionyl spermidine; H*_*2*_*O*_*2*_*: Hydrogen peroxide; HTA: Hemithioacetal; L*^*●*^*: Lipid free radical; LO*_*2*_^*●*^*: Lipid peroxide free radical; LOOH: Lipid peroxide; Lys: Lysine; Met: Methionine; MGO: Methylglyoxal; MGO*^*●-*^*: Methyl glyoxal free radical; NADP: Nicotinamide adenine dinucleotide phosphate; O*_*2*_^*●*^*: Superoxide free radical; ODC: Ornithine decarboxylase; ONOO*^*-*^*: Peroxynitrite; Orn: Ornithine; Put: Putrescine; SAM: S-adenosyl methionine; SAMdc: S-adenosyl methionine decarboxylase; SAMsyn: S-adnosylmethionine Synthase; SDLTSH: S-D-lactoyl trypanothione; SOD: Superoxide dismutase; Spd: Spermidine; SpdS: Spermidine Synthase; T[SH]*_*2*_*: Trypanothione; TDPXo/r: Glutathione peroxidase-like tryparedoxine (oxidized/reduced); TR: Trypanothione reductase; TryP: Tryparedoxin peroxidase; TryS1: Trypanothione synthetase1(Glutathionyl spermidine synthetase); TryS2: Trypanothione synthetase2 (Trypanothione synthetase); TS2: Trypanothione disulphide; TXNo/r: Tryparedoxin (oxidized/reduced); yECS: ϒ-L-glutamyl L-cysteine synthetase*).

**Figure 2:**
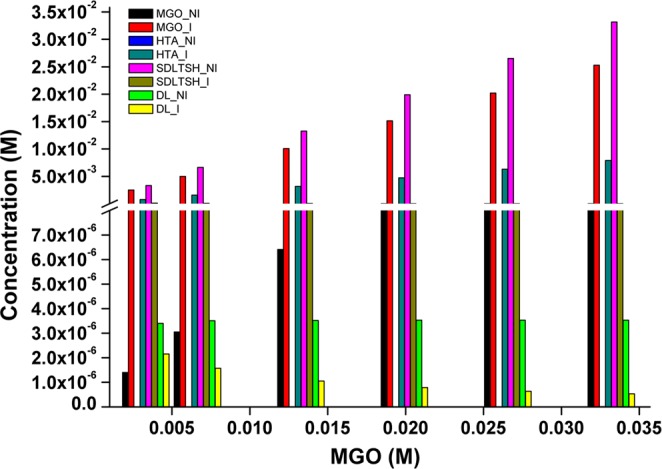
Prediction of the effect of increased conc of MGO in *M1a* and *M1b*.

**Figure 3:**
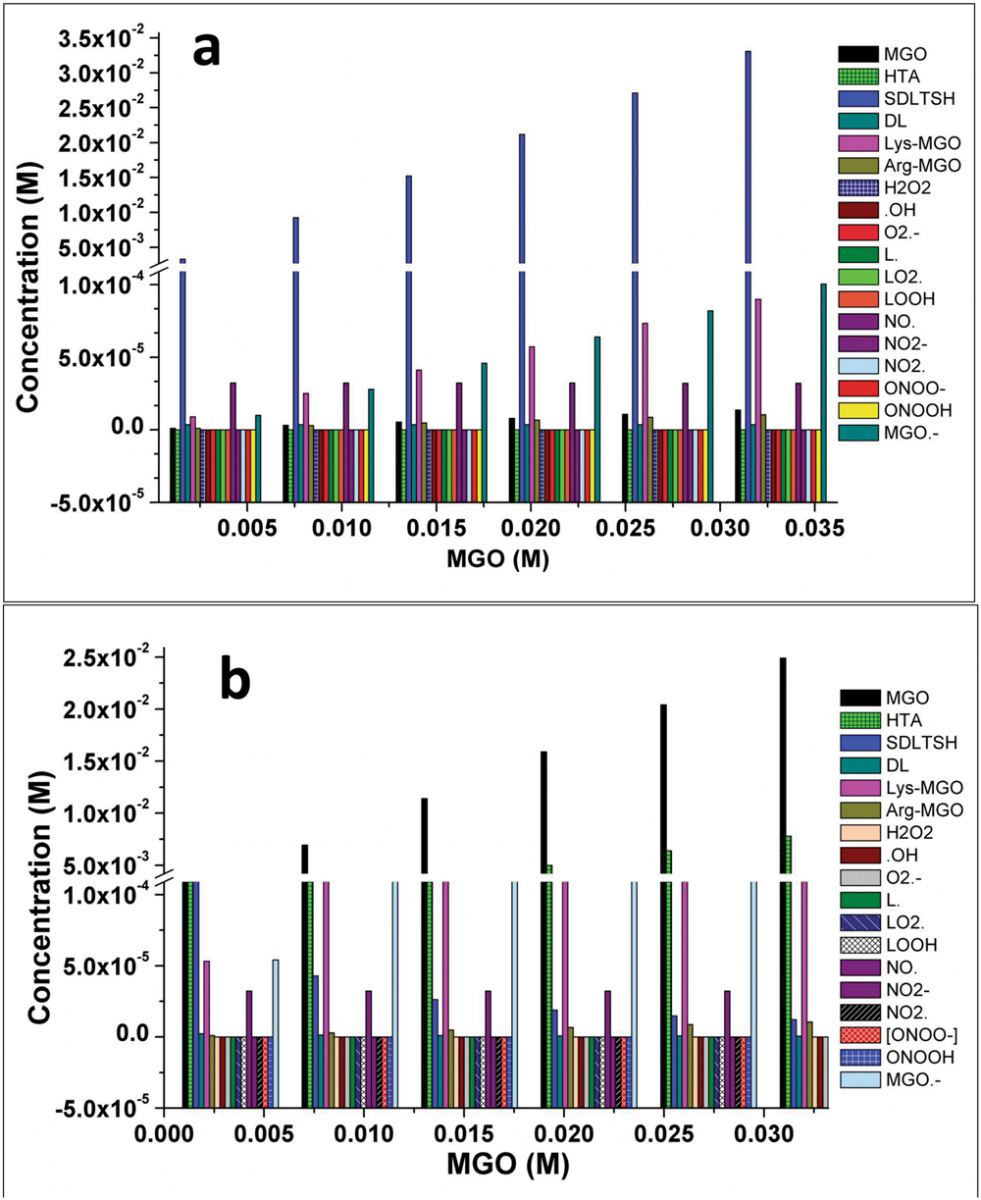
Effect of increased conc of MGO on other metabolites in extended model in *M2a* and *M2b*.

Time course simulation of two models showed maximum activity of GLO I enzyme in terms of the degradation of MGO and synthesis of SDLTSH in *M1a* model when compared to *M1b* model (Figs. 2, S1). The effect of higher concentration of MGO in the system was observed by increasing its initial concentration upto 10 folds. As assumed, increased MGO in *M1b* model has led to the similar level of accumulation of MGO due to the perturbation introduced in the model. This has, consequently, decreased the GLO I activity, thereby, affected the rate of synthesis of SDLTSH and D-lactate (DL) (Fig. 2). It was reported earlier that MGO damages proteins by modifying Arg and Lys residues, forms (AGEs and promotes the formation of free radicals^40–42^. Hence, to understand the effect of MGO accumulation on other metabolites in the system, we extended our models to larger scale and added several reactions (Table 1) of AGEs, in terms of modified Arg and Lys (MGO-Arg and MGO-Lys), and free radical formation (*M*2*a* and *M*2*b*) (Fig. S2). Interestingly, when comparison was made between inhibited and non-inhibited models, the perturbation in extended model demonstrated many folds increase in the formation of many metabolites including MGO-Lys and free radicals (MGO^•-^, ^•^OH, O_2_^•-^, NO_2_^•^) (Fig. 3, Table 3).

**Table 3:** Effect of GLO I inhibition on metabolites produced in inhibited model.

Metabolite	Fold increase^#^
MGO	2212.97x
SDLTSH	0.04x
DL	0.63x
Lys-MGO	5.93x
LO_2_^•^	1.04x
MGO^•-^	5.42x
^•^OH	8.63E + 27x
O_2_^•-^	6.61E + 12x
NO_2_^•^	6.8E + 103x

^#^The concentration of the metabolites produced after introducing the perturbation by GLOI inhibition in the inhibited model were compared with the metabolites concentration obtained in non-inhibited model after kinetic simulation.

**Figure 4:**
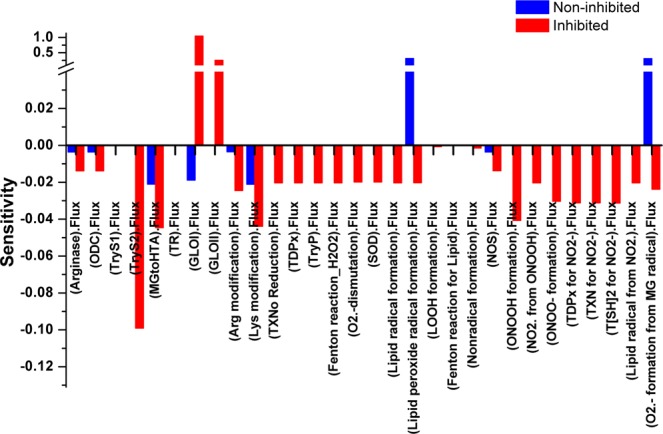
Illustration of Sensitivity analysis of *M2a* (NI: non-inhibited) and *M2b* (I: inhibited) models. For sensitivity analyses in these models, Vmax of GLO I was used to observe its influence on the flux of other reactions. It was observed that reaction fluxes of all reactions were affected in perturbed (inhibited) model.

The robustness analysis, to check the performance and reliability of the kinetic model, was confirmed by varying the values of GLO I reaction (Vmax and Km) (Table S2). Our constructed kinetic model has shown negligible variation in the predicted concentration of SDLTSH. Further, to evaluate the relevance of the GLO I as a potential therapeutic target, sensitivity analysis of the models was performed by using GLOI_Vmax_. It was observed that reaction fluxes of all reactions were found to be affected in perturbed *M*2*b* model (Fig. 4). In *M*2*a* model, most of the reaction were observed independent of GLO I reaction rate as most of the reactions have not shown any sensitivity towards GLOI_Vmax_, however, the perturbation via GLO I reaction demonstrated the opposite suggesting its global impact on reaction fluxes of almost all reactions, thereby, causing the concentration fold increase of the metabolites in *M*2*b*. These observations suggest that free radical formation and other reactions are somehow connected to MGO level and decrease in GLO I activity should affect their formation. Our observations clearly show that the glyoxalase enzyme GLO I is an important therapeutic target. Therefore, it is suggested that GLO I plays an important role in the regulation of redox metabolism by detoxifying MGO. This view is also supported by an earlier study^28^.

### Molecular modelling and pharmacophore features selection

**Figure 5:**
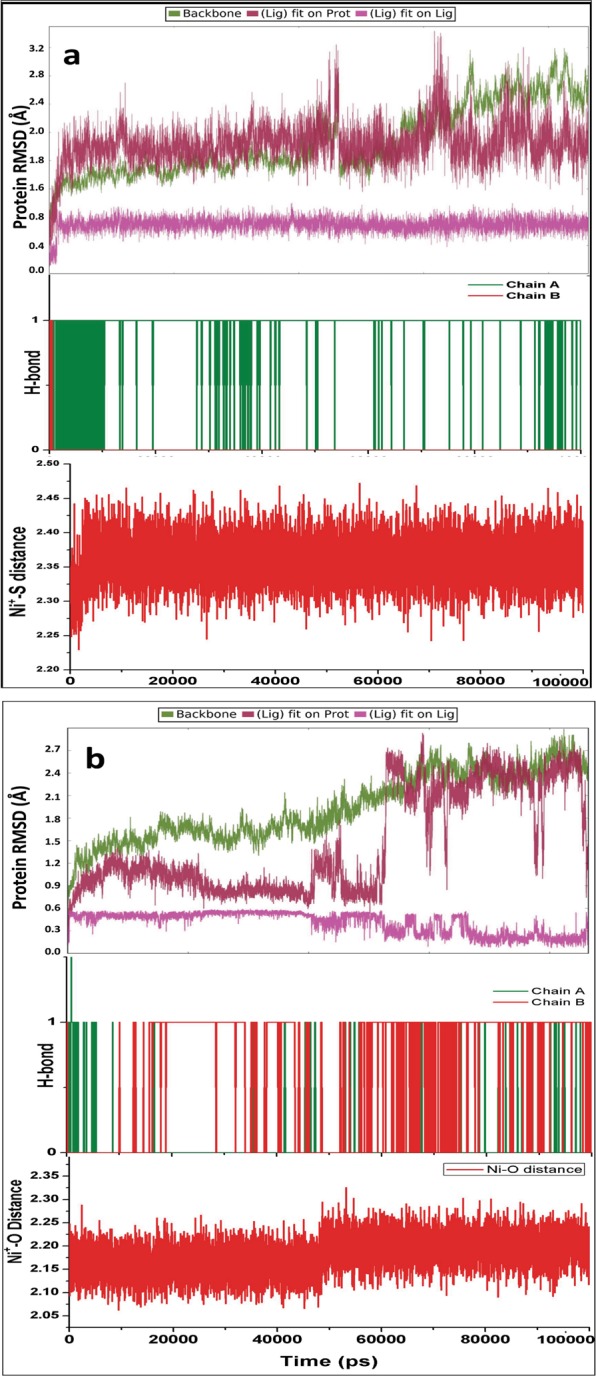
Evaluation of time course simulation of C1 (**a**) and C2 (**b**) with GLO I complex for 100 ns.

**Figure 6:**
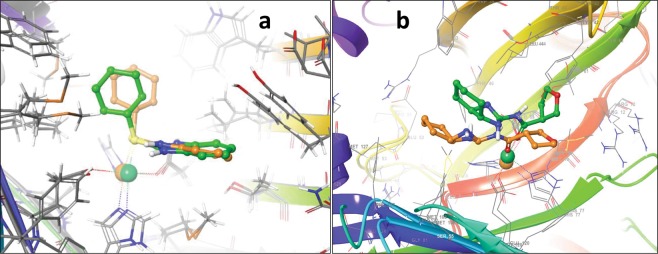
Superposed structures of (**a**) C1- and (**b**) C2-GLOI. ligand and metal ion shown in orange and green color represents structure after 10 ns and 100 ns, respectively.

Benzimidazoles are bicyclic compounds, consisting of fusion of benzene and imidazole, and are known to have potent inhibitory activity^43–46^, including anti-viral, anti-bacterial, anti-leishmanial^47–50^. Similarly, thiosemicarbazones have also been broadly studied due to their role as antibacterial, antiprotozoal, and antiviral activity^51–55^. After ADME/Tox screening and docking, top 8 GLOI-ligand complexes were selected (Table S3) and subjected to MD simulation. Simulation results showed stable interaction between ligands and GLO I active site. RMSD of ligand bound GLO I were plotted to understand their relative stability (Fig. S3). Although, hydrogen bonds plots between ligand-GLO I have demonstrated that all ligands were interacting with the active site by forming at least one H-bond, rmsd in terms of interaction diagram of ligand-GLO I has suggested that only two complexes, C1 and C2 (Benzimidazole derivatives), were stable throughout the simulation cycle without much fluctuation. Furthermore, the ligand distance from Ni^2+^ was observed to be within the range (Fig. S3). These two compound-GLO I complexes were further subjected to extended MD simulation for up to 100 ns to inspect their structural and interaction stability. Our results revealed that only C1-GLO I complex exhibited stable behaviour in terms of rmsd and that C2 tends to deviate from GLO I and fluctuated even after coming into the contact of GLO I (Fig. 5). Same was noted in case of C2-GLO I complex when the two structures from 10 ns and 100 ns were superposed (Fig. 6). Hence, our molecular modelling and simulation analysis suggested that C1 can, further, be used as an active hit against *Leishmania*.

**Figure 7:**
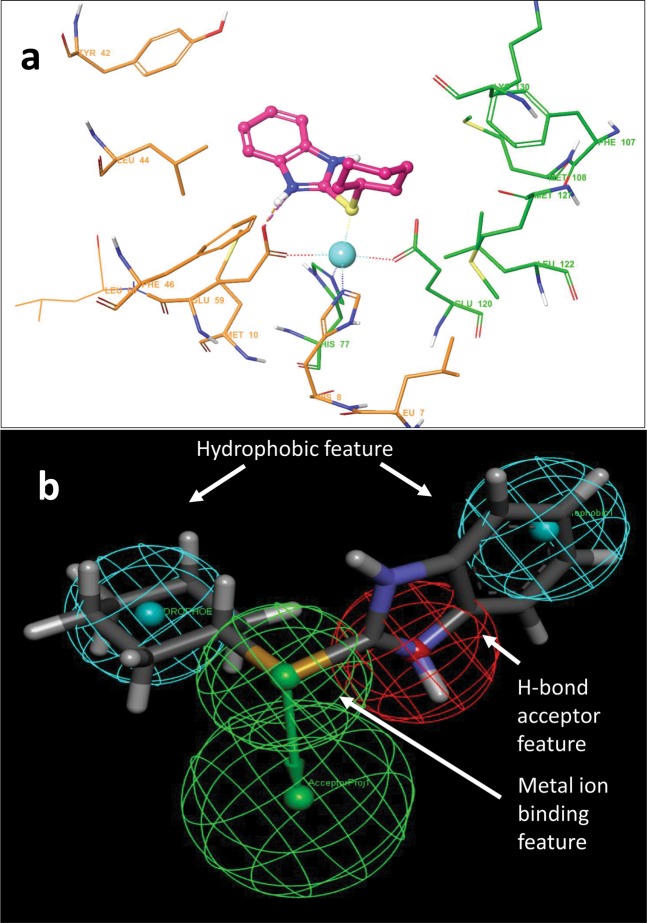
(**a)** Depiction of binding interaction between C1 and GLO I. **(b)** Pharmacophore features selection on the basis of C1-GLO I binding modes. A 5 point pharmacophore model was designed automatically using Discovery studio.

Next, based on C1 binding modes in GLO I active site, initially, a 4-point pharmacophore model was developed using Discovery studio software (Fig. 7). Due to the importance in catalysis^56,57^, the active site metal ion present in the binding pocket of GLO I (Fig. 7a) was manually chosen as ‘metal binding’ feature (Fig. 7b). Finally, then this pharmacophore models was used for database screening against ZINC drug-like database (https://zinc.docking.org/substances/subsets/). The screened compounds were then docked against GLO I. Table S4 shows top 10 compounds selected from pharmacophore based virtual screening.

### Expression and purification of LmGLO I

After reconstitution and transformation of construct (Fig. S4) into BL21 strain, the cells were grown at 37 °C for 24 hrs. The plasmid DNA was isolated and digested by restriction enzyme followed by its gel electrophoresis (Fig. S5a). Further, *gloI* gene was expressed by giving 1 mM IPTG induction for different time point upto 5 h as described in material and methods section (Fig. S5b). The purified GLO I protein through Ni-NTA resin column was found to be ~24 kDa in size^39,58^.

### Anti-promastigote activity of compounds

**Figure 8:**
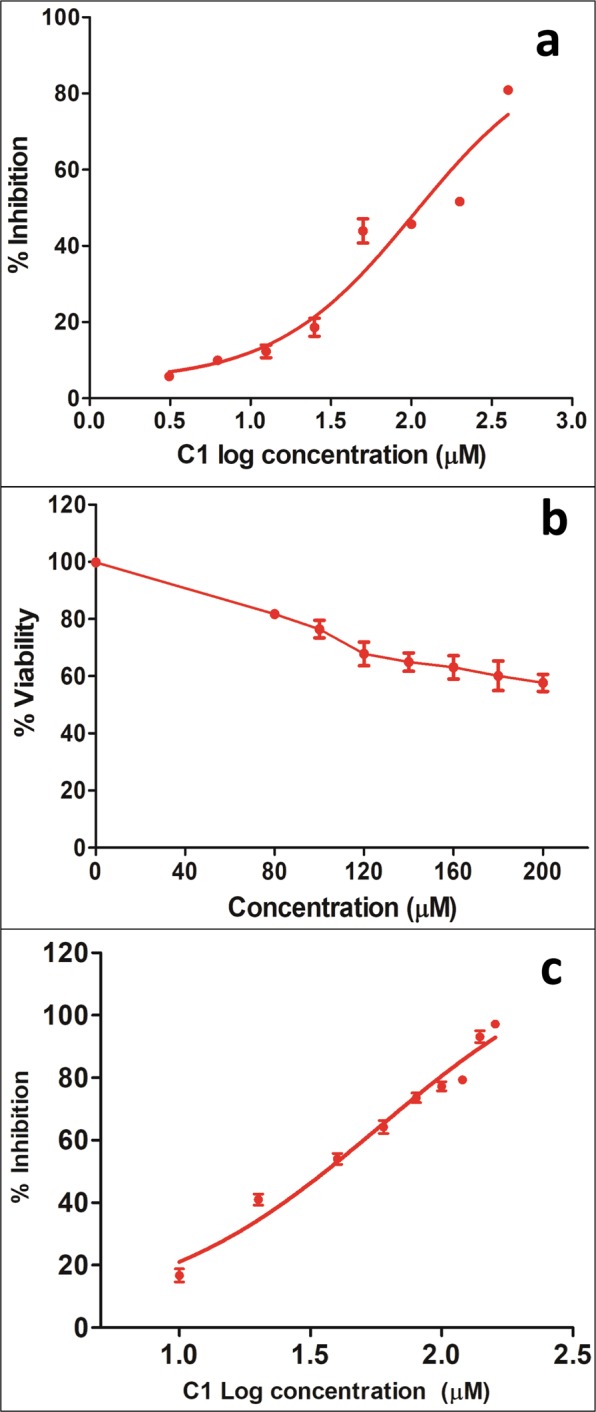
Testing the potency and cytotoxicity of compounds. **(a)** IC50 calculation of C1 against *L. major* parasite. **(b)** Cytotoxicity of the C1 over Raw macrophage cell line. **(c)** IC50 calculation of intracellular *L. major* amastigotes.

**Figure 9:**
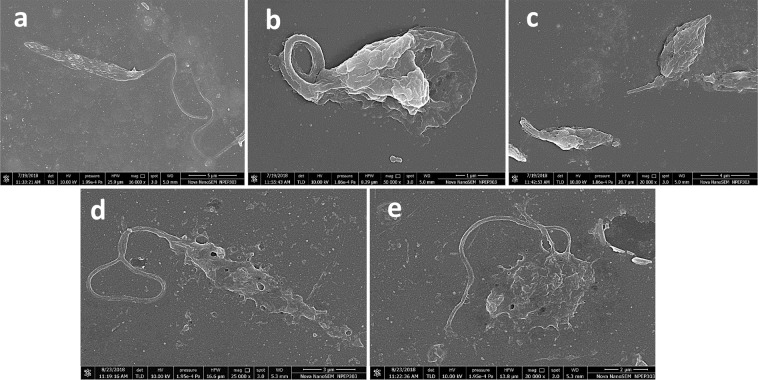
Morphological analysis of untreated and treated Leishmanial promastigotes through SEM. **(a)** untreated, **(b**,**c)** C1 treated, and **(d**,**e)** C2 treated. Both compounds (C1 and C2) caused reduction in size, membrane blebbing and flagella loss causing less motility in the parasite.

Compounds C1 and C2 were procured and tested against *L. major* promastigotes and raw cell line of macrophages post 48 h of treatment and their IC50 values were calculated. Between the two compounds, C2 showed little inhibition and its IC50 against *Leishmania* could not be calculated (IC50 > 1000 μM). However, C1 exhibited promising antileishmanial activity (IC50 105 μM) (Fig. 8a) and ~80% cell viability on macrophage (Fig. 8b). Furthermore, in SEM analysis, promastigotes treated with both the compounds have shown remarkable morphological changes. Both the compounds were able to cause size reduction, membrane blebbing and loss in motility in the parasite (Fig. 9).

### C1 efficiently eliminates *L. major* amastigotes from macrophage

C1 has also shown effective parasite clearance property in case of intracellular amastigotes (Fig. 8c). It was observed that C1 was more effective and exhibited slightly higher potency against the intracellular amastigotes (IC_50_ 57.64 µM) compared to *L. major* promastigotes (IC50 105 µM).

### C1 induces sub-G0/G1 phase cell-division arrest and phosphatidylserine externalization

**Figure 10:**
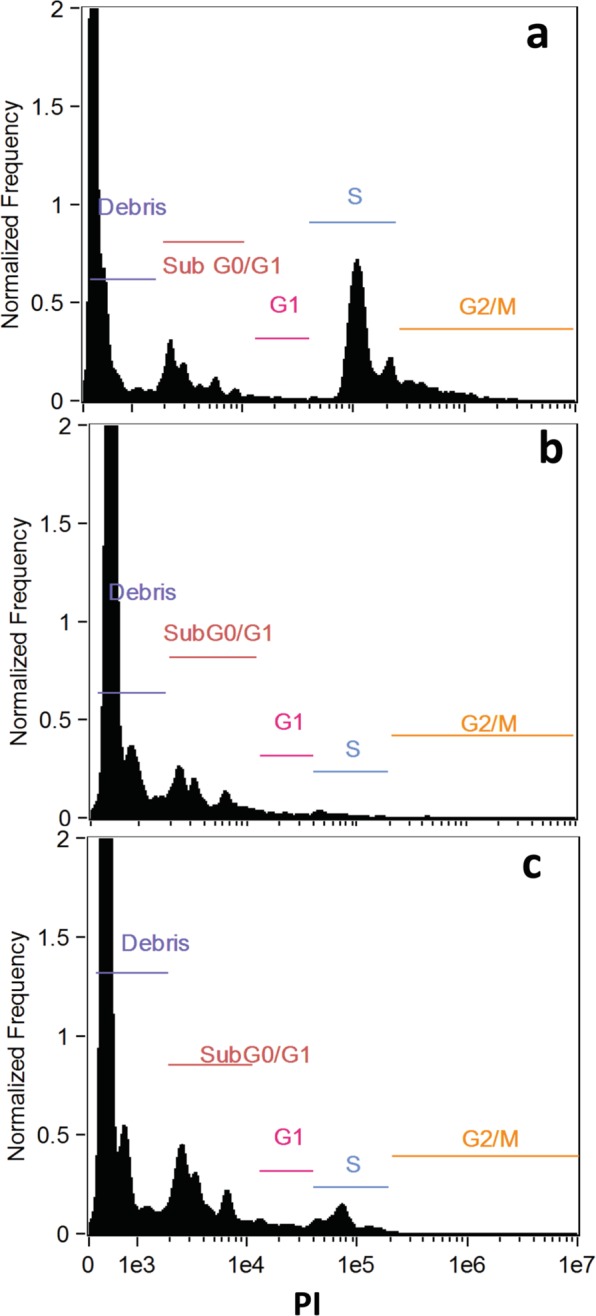
Cell cycle analysis of *L. major*. (**a**) Untreated; and treated (**b**) MIL; (**c**) C1.

**Figure 11:**
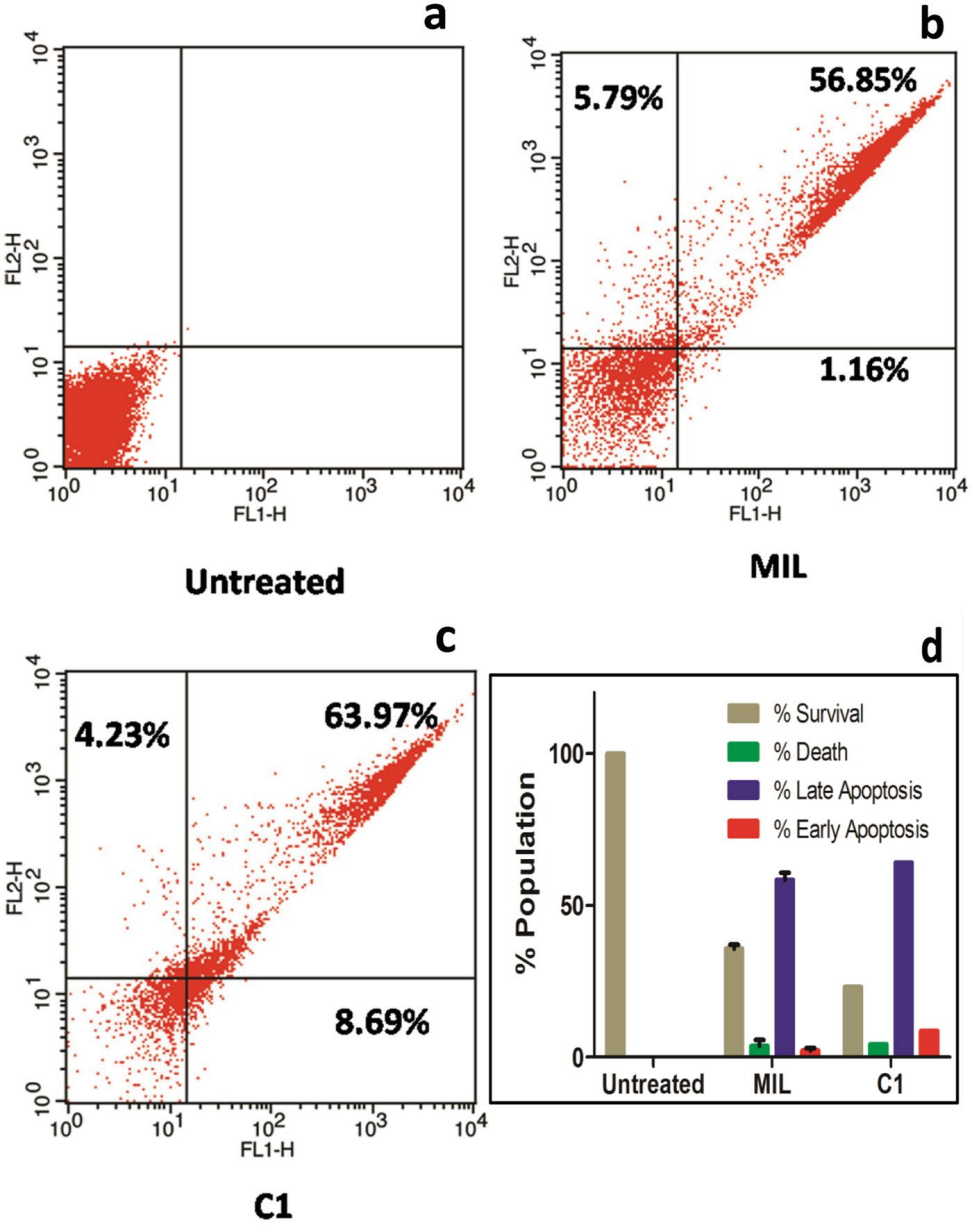
Apoptosis analysis of untreated and treated (MIL and C1) *L. major*.

Our results revealed that C1 treated promastigotes showed 17.12% increase in sub-G0/G1 phase when compared with untreated parasites (Fig. 10). This increase was accompanied by decrease in the number of cells in S and G2/M phase (4.78% and 0.11%, respectively) in comparison to untreated cells (18.57% and 4.95%, respectively). While in case of MIL treated parasite lower values were obtained (sub-G0/G1: 10.19%, S: 1.06%, and G2/M: 0.18%). These findings suggest that C1 arrests parasites in sub-G0/G1 phase and induces cell death resembling apoptosis^59–63^. We could also observe that approximately 8.69% treated parasite population was annexinV-positive and PI-negative (Fig. 11). Therefore, the anti-leishmanial activity of C1 was observed as apoptosis driven due to the lower percentage of PI-positive population (4.23%). Similarly, benzimidazoles have reported to cause cell cycle arrest and trigger apoptosis-like phenomenon in *Leishmania*^64,65^. Taken together, increased proportion of cells arrested in sub-G0/G1 phase and higher early apoptotic percentage confirmed that C1 induces apoptosis in *Leishmanial* promastigotes.

### Efficacy of C1 in L. major infected mice

**Figure 12:**
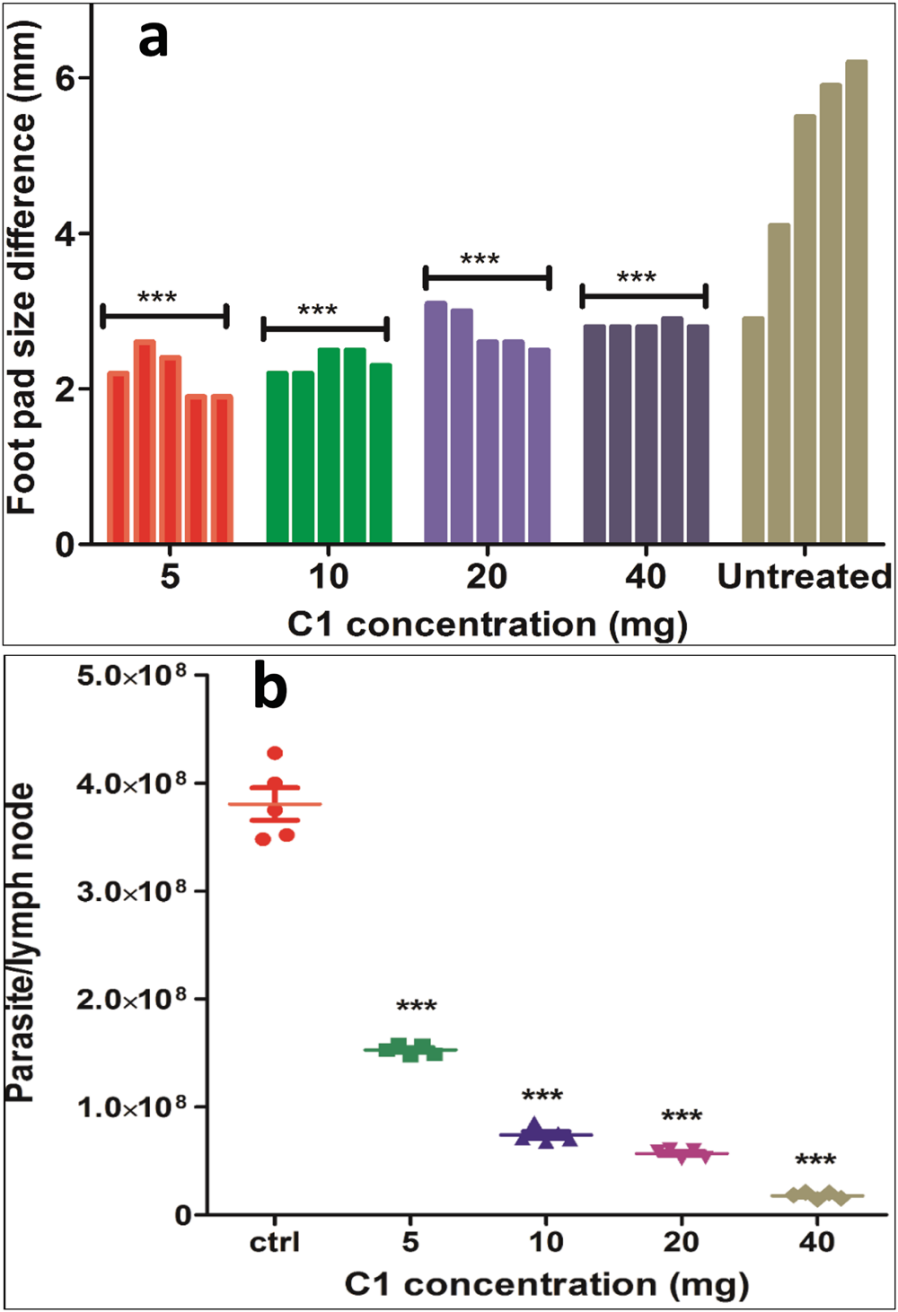
(**a)** Foot pad size difference in C1 treated and untreated groups. **(b)** Parasite load assay of the C1 treated and untreated groups. Every dot indicates one mice. P-value was calculated by comparing treated groups with control and significance is indicated in the figure as ***p < 0.0001.

As mentioned above, the drug C1 was administered orally in different groups (5, 10, 20 and 40 mg/kg/body weight/day). The swelling in the foot pad was monitored post treatment and it was observed that in the groups who received C1 did not show any difference in the lesion size unlike the control group administered with water showing gradual increase in lesion size (Fig. 12a). To confirm the efficacy of C1, treated and untreated mice were euthanized and their lymph nodes were collected to find parasitic burden. It was noted that in all treated groups the parasitic load found to be decreased as compared top untreated mice (Fig. 12b). Moreover, it was noted that at higher dose (40 mg) the compound showed little toxic effect that caused weakening of mice. Our results indicate that C1 has healing effect in infected mice and reduced the parasitic burden in mice. Henceforth, we suggest C1 as potent anti-leishmanial hit and by further modification it could be possible to develop novel and potent compounds.

### Glyoxalase I assay

**Figure 13:**
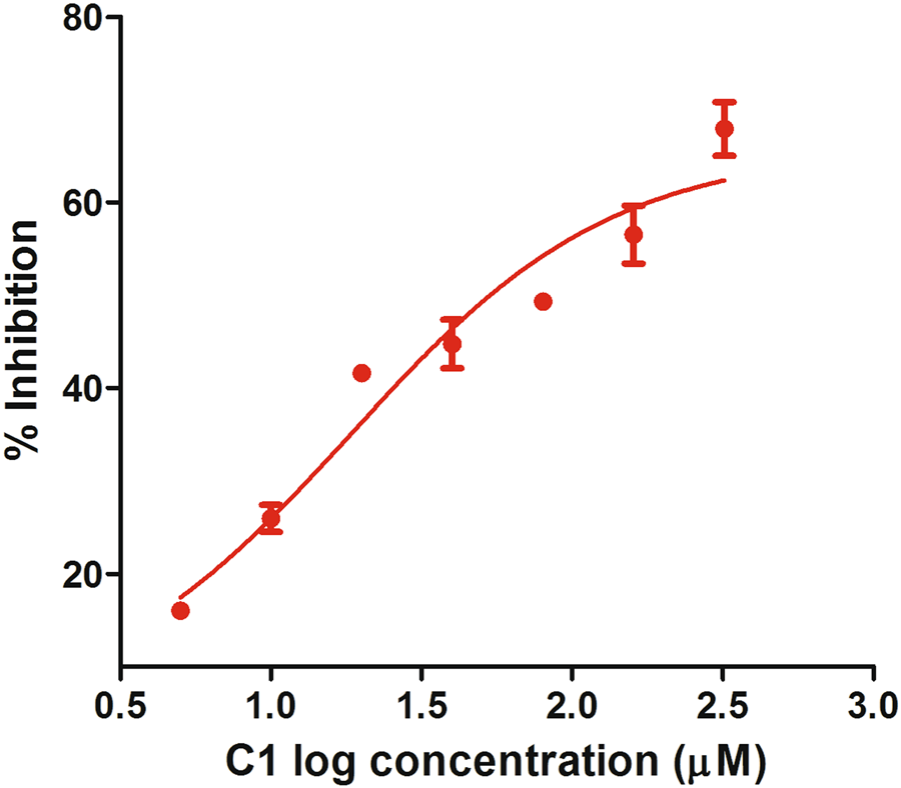
Illustration of inhibitory potency of C1 against LmGLOI.

An IC50 of 18.34 µM for C1 against LmGLOI was obtained after the data fitting to sigmoidal dose-response curves with nonlinear regression in GraphPad Prism 5 (Fig. 13). To see the potency of C1, this IC50 was further converted to *Ki* value using the Cheng-Prusoff equation using online IC50 to *Ki* conversion tool^66^. For this published Km value of LmGLOI was used. Our Ki value for C1 (2.52 µM) was found to be lower than that of the previously investigated inhibitors against LmGLOI^67^ suggesting the potency of C1 against LmGLOI.

## Conclusion

**Figure 14:**
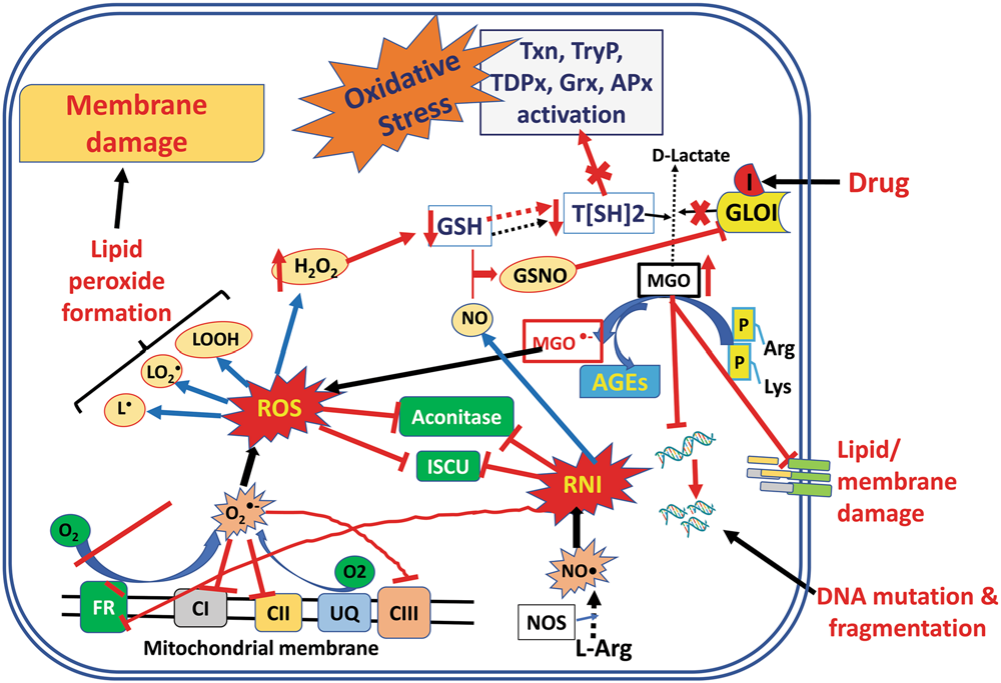
Depiction of generation and effect of free radicals. Inside mitochondria, source of generation of ROS (reactive oxygen species) are Fumarase reductase (FR) and Ubiquinone 9 (UQ) carrying single electron reaction in ETC in mitochondrial membrane contributing to the generation of superoxide anion free radicals (**O**_**2**_^**•−**^) which are usually detoxified by other membrane bound complexes (eg., cytc, etc., not shown in the figure) by transporting the electron to oxygen. Superoxide anion free radicals contribute to form other ROS and damage various proteins, DNA and lipids. Similarly, leishmanial Nitric oxide synthase (NOS), in unfavorable circumstances, synthesizes **NO•** ions that further contribute to the formation of RNI (reactive nitrogen species). ROS and RNI also inactivate Fe-S cluster proteins (Fumarase reductase (FR) and Aconitase) by leaching out their iron centres. ROS and RNI also decrease GSH (glutathione) level thereby directly affecting the synthesis of trypanothione (T[SH]_2_) and activation of various T[SH]_2_ dependent proteins and enzymes. These reactive species also inactivates glyoxalase I (GLOI), an important rate limiting enzyme in a two-step methylglyoxal (MGO), a highly reactive metabolite and a by-product of glycolysis, detoxification pathway. Further, inactivation of GLOI enzyme can also be carried out by drugs or inhibitors leading to the increased level of MGO. MGO, when in excess, irreversibly modifies proteins (including GLOI) by reacting with their Arg and Lys residues and forms advanced glycation end products (AGEs), (leading to the degradation of proteins), and MGO^**•-**^ free radicals.

Taken together, our study demonstrates the amalgamation of systems biology and structural modelling to understand the effect of GLO I inhibition kinetics and to use this knowledge into exploring potent lead compounds. Our model reveals that the perturbation introduced in the form of GLO I inhibition had noticeable effect on MGO detoxification that ultimately led to increased formation of free radicals (Fig. 14). Moreover, via molecular modelling we were able to screen C1 as hit compound that also exhibited good anti-leishmanial activity *in vitro*. This compound caused morphological alteration in the parasite and was also found to induce cell cycle arrest and apoptosis in *L. major*. Furthermore, *in vivo* studies on mice model have revealed the parasite clearance potency of the compound and proved the overall efficacy of C1.

## Supplementary information

Supplementary file.
