# Supplementary material for: Multiscale Process Modelling in Translational Systems Biology of Leishmania major: A Holistic view
Source: Sci Rep. 2020 Jan 21;10:785. doi: 10.1038/s41598-020-57640-4 (PMC6972910; doi:10.1038/s41598-020-57640-4)
Supplement: Supplementary file 1 — Supplementary file. [file 41598_2020_57640_MOESM1_ESM.pdf]

Supplementary file_1

Multiscale Process Modelling in Translational Systems Biology of *Leishmania major*: A Holistic view

Nutan Chauhan, Shailza Singh

$$\frac{d([Met]\cdot V_{cell})}{dt} = -V_{cell}\cdot\left(\frac{3.4e\text{-}009\cdot[Met]^{2.3}}{0.00025^{2.3}+[Met]^{2.3}}\right)$$

$$\frac{d([SAM]\cdot V_{cell})}{dt} = +V_{cell}\cdot\left(\frac{3.4e\text{-}009\cdot[Met]^{2.3}}{0.00025^{2.3}+[Met]^{2.3}}\right) - V_{cell}\cdot\left(\frac{5.2e\text{-}009\cdot[SAM]}{0.00038+[SAM]}\right)$$

$$\frac{d([dcSAM]\cdot V_{cell})}{dt} = +V_{cell}\cdot\left(\frac{5.2e\text{-}009\cdot[SAM]}{0.00038+[SAM]}\right) - V_{cell}\cdot\left(\frac{1.98e\text{-}013\cdot[Put]\cdot[dcSAM]}{9e\text{-}008\cdot[Put]+0.000205\cdot[dcSAM]+[Put]\cdot[dcSAM]+0.1\cdot 0.000205}\right)$$

$$\frac{d([Arg]\cdot V_{cell})}{dt} = -V_{cell}\cdot(0.0085\cdot[Arg]\cdot[MGO]) - V_{cell}\cdot\left(\frac{0.0024\cdot[Arg]}{0.0215+[Arg]}\right) - V_{cell}\cdot\left(\frac{3.5e\text{-}006\cdot[Arg]\cdot[NADPH]}{7e\text{-}007\cdot[Arg]+4.9e\text{-}006\cdot[NADPH]+[Arg]\cdot[NADPH]+0.1\cdot 7e\text{-}007}\right)$$

$$\frac{d([Orn]\cdot V_{cell})}{dt} = +V_{cell}\cdot\left(\frac{0.0024\cdot[Arg]}{0.0215+[Arg]}\right) - V_{cell}\cdot\left(\frac{0.025256\cdot[Orn]}{0.00042+[Orn]}\right)$$

$$\frac{d([Put]\cdot V_{cell})}{dt} = +V_{cell}\cdot\left(\frac{0.025256\cdot[Orn]}{0.00042+[Orn]}\right) - V_{cell}\cdot\left(\frac{1.98e\text{-}013\cdot[Put]\cdot[dcSAM]}{9e\text{-}008\cdot[Put]+0.000205\cdot[dcSAM]+[Put]\cdot[dcSAM]+0.1\cdot 0.000205}\right)$$

$$\frac{d([Spd]\cdot V_{cell})}{dt} = +V_{cell}\cdot\left(\frac{1.98e\text{-}013\cdot[Put]\cdot[dcSAM]}{9e\text{-}008\cdot[Put]+0.000205\cdot[dcSAM]+[Put]\cdot[dcSAM]+0.1\cdot 0.000205}\right) - V_{cell}\cdot\left(\frac{8e\text{-}007\cdot[Spd]\cdot[GSH]}{0.00094\cdot 8.9e\text{-}005+[Spd]\cdot 8.9e\text{-}005+[GSH]\cdot 0.00094+[Spd]\cdot[GSH]}\right)$$

$$\frac{d([Cys]\cdot V_{cell})}{dt} = -V_{cell}\cdot\left(\frac{1.8e\text{-}006\cdot[Cys]\cdot[Glu]}{0.0017\cdot[Cys]+0.0092\cdot[Glu]+[Cys]\cdot[Glu]+9.37e\text{-}005\cdot 0.0017}\right)$$

$$\frac{d([Glu]\cdot V_{cell})}{dt} = -V_{cell}\cdot\left(\frac{1.8e\text{-}006\cdot[Cys]\cdot[Glu]}{0.0017\cdot[Cys]+0.0092\cdot[Glu]+[Cys]\cdot[Glu]+9.37e\text{-}005\cdot 0.0017}\right)$$

$$\frac{d([GluCys]\cdot V_{cell})}{dt} = +V_{cell}\cdot\left(\frac{1.8e\text{-}006\cdot[Cys]\cdot[Glu]}{0.0017\cdot[Cys]+0.0092\cdot[Glu]+[Cys]\cdot[Glu]+9.37e\text{-}005\cdot 0.0017}\right) - V_{cell}\cdot\left(\frac{3.4e\text{-}008\cdot[Gly]\cdot[GluCys]}{0.0012\cdot[Gly]+4e\text{-}005\cdot[GluCys]+[Gly]\cdot[GluCys]+8e\text{-}005\cdot 0.0012}\right)$$

$$\frac{d([GSH]\cdot V_{cell})}{dt} = +V_{cell}\cdot\left(\frac{3.4e\text{-}008\cdot[Gly]\cdot[GluCys]}{0.0012\cdot[Gly]+4e\text{-}005\cdot[GluCys]+[Gly]\cdot[GluCys]+8e\text{-}005\cdot 0.0012}\right) - V_{cell}\cdot\left(\frac{8e\text{-}007\cdot[Spd]\cdot[GSH]}{0.00094\cdot 8.9e\text{-}005+[Spd]\cdot 8.9e\text{-}005+[GSH]\cdot 0.00094+[Spd]\cdot[GSH]}\right) - V_{cell}\cdot\left(\frac{8e\text{-}007\cdot[Gspd]\cdot[GSH]}{4e\text{-}005\cdot 8.9e\text{-}005+[Gspd]\cdot 8.9e\text{-}005+[GSH]\cdot 4e\text{-}005+[Gspd]\cdot[GSH]}\right)$$

$$\frac{d([Gly] \cdot V_{cell})}{d\,t} = -V_{cell} \cdot \left(\frac{3.4e\text{-}008 \cdot [Gly] \cdot [GluCys]}{0.0012 \cdot [Gly] + 4e\text{-}005 \cdot [GluCys] + [Gly] \cdot [GluCys] + 8e\text{-}005 \cdot 0.0012}\right)$$

$$\frac{d([Gspd] \cdot V_{cell})}{d\,t} = +V_{cell} \cdot \left(\frac{8e\text{-}007 \cdot [Spd] \cdot [GSH]}{0.00094 \cdot 8.9e\text{-}005 + [Spd] \cdot 8.9e\text{-}005 + [GSH] \cdot 0.00094 + [Spd] \cdot [GSH]}\right)$$
$$-V_{cell} \cdot \left(\frac{8e\text{-}007 \cdot [Gspd] \cdot [GSH]}{4e\text{-}005 \cdot 8.9e\text{-}005 + [Gspd] \cdot 8.9e\text{-}005 + [GSH] \cdot 4e\text{-}005 + [Gspd] \cdot [GSH]}\right)$$

$$\frac{d([MGO] \cdot V_{cell})}{d\,t} = +V_{cell} \cdot (0.016 \cdot [HTA])$$
$$-V_{cell} \cdot (0.0085 \cdot [Arg] \cdot [MGO])$$
$$-V_{cell} \cdot (0.0068 \cdot [Lys] \cdot [MGO])$$
$$+V_{cell} \cdot (1e\text{+}006 \cdot [LO2.] \cdot [LO2.])$$
$$+V_{cell} \cdot (1.31e\text{-}010 \cdot [\text{"MGO.-"}] \cdot [O2])$$
$$-V_{cell} \cdot (0.0056 \cdot [MGO] \cdot [T[SH]2])$$

$$\frac{d([HTA] \cdot V_{cell})}{d\,t} = -V_{cell} \cdot (0.016 \cdot [HTA])$$
$$-V_{cell} \cdot \left(\frac{0.000159 \cdot [HTA]}{3.2e\text{-}005 + [HTA]}\right)$$
$$+V_{cell} \cdot (0.0056 \cdot [MGO] \cdot [T[SH]2])$$

$$\frac{d([DL] \cdot V_{cell})}{d\,t} = +V_{cell} \cdot \left(\frac{2.3e\text{-}009 \cdot [SDLTSH]}{3.9e\text{-}005 + [SDLTSH]}\right)$$

$$\frac{d([NADP] \cdot V_{cell})}{d\,t} = +V_{cell} \cdot \left(\frac{3.33e\text{-}006 \cdot [TS2] \cdot [NADPH]}{2e\text{-}005 \cdot [TS2] + 5e\text{-}005 \cdot [NADPH] + [TS2] \cdot [NADPH] + 0.1 \cdot 2e\text{-}005}\right)$$
$$+V_{cell} \cdot \left(\frac{3.5e\text{-}006 \cdot [Arg] \cdot [NADPH]}{7e\text{-}007 \cdot [Arg] + 4.9e\text{-}006 \cdot [NADPH] + [Arg] \cdot [NADPH] + 0.1 \cdot 7e\text{-}007}\right)$$

$$\frac{d([NADPH] \cdot V_{cell})}{d\,t} = -V_{cell} \cdot \left(\frac{3.33e\text{-}006 \cdot [TS2] \cdot [NADPH]}{2e\text{-}005 \cdot [TS2] + 5e\text{-}005 \cdot [NADPH] + [TS2] \cdot [NADPH] + 0.1 \cdot 2e\text{-}005}\right)$$
$$-V_{cell} \cdot \left(\frac{3.5e\text{-}006 \cdot [Arg] \cdot [NADPH]}{7e\text{-}007 \cdot [Arg] + 4.9e\text{-}006 \cdot [NADPH] + [Arg] \cdot [NADPH] + 0.1 \cdot 7e\text{-}007}\right)$$

$$\frac{d([TS2] \cdot V_{cell})}{d\,t} = -V_{cell} \cdot \left(\frac{3.33e\text{-}006 \cdot [TS2] \cdot [NADPH]}{2e\text{-}005 \cdot [TS2] + 5e\text{-}005 \cdot [NADPH] + [TS2] \cdot [NADPH] + 0.1 \cdot 2e\text{-}005}\right)$$
$$+V_{cell} \cdot (200 \cdot [T[SH]2] \cdot [TXNo])$$
$$+V_{cell} \cdot (7200 \cdot [T[SH]2] \cdot [\text{"ONOO-"}])$$

$$\frac{d([T[SH]2] \cdot V_{cell})}{dt} = +V_{cell} \cdot \left(\frac{3.33e\text{-}006 \cdot [TS2] \cdot [NADPH]}{2e\text{-}005 \cdot [TS2] + 5e\text{-}005 \cdot [NADPH] + [TS2] \cdot [NADPH] + 0.1 \cdot 2e\text{-}005}\right)$$
$$+V_{cell} \cdot (0.016 \cdot [HTA])$$
$$+V_{cell} \cdot \left(\frac{2.3e\text{-}009 \cdot [SDLTSH]}{3.9e\text{-}005 + [SDLTSH]}\right)$$
$$-V_{cell} \cdot (200 \cdot [T[SH]2] \cdot [TXNo])$$
$$-V_{cell} \cdot (7200 \cdot [T[SH]2] \cdot ["ONOO-"])$$
$$+V_{cell} \cdot \left(\frac{8e\text{-}007 \cdot [Gspd] \cdot [GSH]}{4e\text{-}005 \cdot 8.9e\text{-}005 + [Gspd] \cdot 8.9e\text{-}005 + [GSH] \cdot 4e\text{-}005 + [Gspd] \cdot [GSH]}\right)$$
$$-V_{cell} \cdot (0.0056 \cdot [MGO] \cdot [T[SH]2])$$

$$\frac{d([CO2] \cdot V_{cell})}{dt} = +V_{cell} \cdot \left(\frac{0.025256 \cdot [Orn]}{0.00042 + [Orn]}\right)$$

$$\frac{d([SDLTSH] \cdot V_{cell})}{dt} = +V_{cell} \cdot \left(\frac{0.000159 \cdot [HTA]}{3.2e\text{-}005 + [HTA]}\right)$$
$$-V_{cell} \cdot \left(\frac{2.3e\text{-}009 \cdot [SDLTSH]}{3.9e\text{-}005 + [SDLTSH]}\right)$$

$$\frac{d(["Arg-MG"] \cdot V_{cell})}{dt} = +V_{cell} \cdot (0.0085 \cdot [Arg] \cdot [MGO])$$

$$\frac{d([Lys] \cdot V_{cell})}{dt} = -V_{cell} \cdot (0.0068 \cdot [Lys] \cdot [MGO])$$

$$\frac{d(["Lys-MG"] \cdot V_{cell})}{dt} = +V_{cell} \cdot (0.0068 \cdot [Lys] \cdot [MGO])$$

$$\frac{d([TXNo] \cdot V_{cell})}{dt} = -V_{cell} \cdot (200 \cdot [T[SH]2] \cdot [TXNo])$$
$$+V_{cell} \cdot \left(\frac{15.4 \cdot [TDPx] \cdot [H2O2] \cdot [TXNr]}{2.2e\text{-}006 \cdot [H2O2] + 0.000193 \cdot [TXNr] + [H2O2] \cdot [TXNr]}\right)$$
$$+V_{cell} \cdot \left(\frac{8.8 \cdot [TryP] \cdot [H2O2] \cdot [TXNr]}{4.9e\text{-}006 \cdot [H2O2] + 6.3e\text{-}006 \cdot [TXNr] + [H2O2] \cdot [TXNr]}\right)$$
$$+V_{cell} \cdot (220000 \cdot [TXNr] \cdot [TDPxo])$$
$$+V_{cell} \cdot (3500 \cdot [TXNr] \cdot ["ONOO-"])$$

$$\frac{d([TXNr] \cdot V_{cell})}{dt} = +V_{cell} \cdot (200 \cdot [T[SH]2] \cdot [TXNo])$$
$$-V_{cell} \cdot \left(\frac{15.4 \cdot [TDPx] \cdot [H2O2] \cdot [TXNr]}{2.2e\text{-}006 \cdot [H2O2] + 0.000193 \cdot [TXNr] + [H2O2] \cdot [TXNr]}\right)$$
$$-V_{cell} \cdot \left(\frac{8.8 \cdot [TryP] \cdot [H2O2] \cdot [TXNr]}{4.9e\text{-}006 \cdot [H2O2] + 6.3e\text{-}006 \cdot [TXNr] + [H2O2] \cdot [TXNr]}\right)$$
$$-V_{cell} \cdot (220000 \cdot [TXNr] \cdot [TDPxo])$$
$$-V_{cell} \cdot (3500 \cdot [TXNr] \cdot ["ONOO-"])$$

$$\frac{d([H2O] \cdot V_{cell})}{dt} = +V_{cell} \cdot \left(\frac{15.4 \cdot [TDPx] \cdot [H2O2] \cdot [TXNr]}{2.2e\text{-}006 \cdot [H2O2] + 0.000193 \cdot [TXNr] + [H2O2] \cdot [TXNr]}\right)$$
$$+V_{cell} \cdot \left(\frac{8.8 \cdot [TryP] \cdot [H2O2] \cdot [TXNr]}{4.9e\text{-}006 \cdot [H2O2] + 6.3e\text{-}006 \cdot [TXNr] + [H2O2] \cdot [TXNr]}\right)$$
$$+V_{cell} \cdot (1e{+}010 \cdot [.OH] \cdot [LH])$$
$$+V_{cell} \cdot (100000 \cdot [ONOOH] \cdot [H2O2])$$

$$\frac{d([H2O2]\cdot V_{cell})}{dt} = -V_{cell}\cdot\left(\frac{15.4\cdot[TDPx]\cdot[H2O2]\cdot[TXNr]}{2.2e\text{-}006\cdot[H2O2]+0.000193\cdot[TXNr]+[H2O2]\cdot[TXNr]}\right)$$
$$-V_{cell}\cdot\left(\frac{8.8\cdot[TryP]\cdot[H2O2]\cdot[TXNr]}{4.9e\text{-}006\cdot[H2O2]+6.3e\text{-}006\cdot[TXNr]+[H2O2]\cdot[TXNr]}\right)$$
$$-V_{cell}\cdot(76\cdot[H2O2]\cdot[Fe2])$$
$$+V_{cell}\cdot(500000\cdot["O2.-"]\cdot["O2.-"]\cdot["H+"])$$
$$+V_{cell}\cdot(1.6e+009\cdot["O2.-"]\cdot["O2.-"])$$
$$-V_{cell}\cdot(100000\cdot[ONOOH]\cdot[H2O2])$$

$$\frac{d([.OH]\cdot V_{cell})}{dt} = +V_{cell}\cdot(76\cdot[H2O2]\cdot[Fe2])$$
$$-V_{cell}\cdot(1e+010\cdot[.OH]\cdot[LH])$$
$$-V_{cell}\cdot(1.4e+010\cdot[NO2.]\cdot[.OH])$$

$$\frac{d([Fe2]\cdot V_{cell})}{dt} = -V_{cell}\cdot(76\cdot[H2O2]\cdot[Fe2])$$
$$-V_{cell}\cdot(10000\cdot[LOOH]\cdot[Fe2])$$

$$\frac{d([Fe3]\cdot V_{cell})}{dt} = +V_{cell}\cdot(76\cdot[H2O2]\cdot[Fe2])$$
$$+V_{cell}\cdot(10000\cdot[LOOH]\cdot[Fe2])$$

$$\frac{d(["O2.-"]\cdot V_{cell})}{dt} = -2\cdot V_{cell}\cdot(500000\cdot["O2.-"]\cdot["O2.-"]\cdot["H+"])$$
$$-2\cdot V_{cell}\cdot(1.6e+009\cdot["O2.-"]\cdot["O2.-"])$$
$$+V_{cell}\cdot(100000\cdot[ONOOH]\cdot[H2O2])$$
$$-V_{cell}\cdot(3.4e+007\cdot[NO2.]\cdot["O2.-"])$$
$$+V_{cell}\cdot(1.31e\text{-}010\cdot["MGO.-"]\cdot[O2])$$

$$\frac{d([O2]\cdot V_{cell})}{dt} = +V_{cell}\cdot(500000\cdot["O2.-"]\cdot["O2.-"]\cdot["H+"])$$
$$+V_{cell}\cdot(1.6e+009\cdot["O2.-"]\cdot["O2.-"])$$
$$-V_{cell}\cdot(3e+008\cdot[L.]\cdot[O2])$$
$$-V_{cell}\cdot(1.31e\text{-}010\cdot["MGO.-"]\cdot[O2])$$

$$\frac{d([L.]\cdot V_{cell})}{dt} = +V_{cell}\cdot(1e+010\cdot[.OH]\cdot[LH])$$
$$-V_{cell}\cdot(3e+008\cdot[L.]\cdot[O2])$$
$$+V_{cell}\cdot(50\cdot[LO2.]\cdot[LH])$$
$$+V_{cell}\cdot(1e+006\cdot[NO2.]\cdot[LH])$$

$$\frac{d([LH]\cdot V_{cell})}{dt} = -V_{cell}\cdot(1e+010\cdot[.OH]\cdot[LH])$$
$$-V_{cell}\cdot(50\cdot[LO2.]\cdot[LH])$$
$$-V_{cell}\cdot(1e+006\cdot[NO2.]\cdot[LH])$$

$$\frac{d([LO2.]\cdot V_{cell})}{dt} = +V_{cell}\cdot(3e{+}008\cdot[L.]\cdot[O2]) \\ -V_{cell}\cdot(50\cdot[LO2.]\cdot[LH]) \\ +V_{cell}\cdot(10000\cdot[LOOH]\cdot[Fe2]) \\ -2\cdot V_{cell}\cdot(1e{+}006\cdot[LO2.]\cdot[LO2.])$$

$$\frac{d([LOOH]\cdot V_{cell})}{dt} = +V_{cell}\cdot(50\cdot[LO2.]\cdot[LH]) \\ -V_{cell}\cdot(10000\cdot[LOOH]\cdot[Fe2])$$

$$\frac{d([Citrulline]\cdot V_{cell})}{dt} = +V_{cell}\cdot\left(\frac{3.5e{-}006\cdot[Arg]\cdot[NADPH]}{7e{-}007\cdot[Arg]+4.9e{-}006\cdot[NADPH]+[Arg]\cdot[NADPH]+0.1\cdot 7e{-}007}\right)$$

$$\frac{d([NO.]\cdot V_{cell})}{dt} = +V_{cell}\cdot\left(\frac{3.5e{-}006\cdot[Arg]\cdot[NADPH]}{7e{-}007\cdot[Arg]+4.9e{-}006\cdot[NADPH]+[Arg]\cdot[NADPH]+0.1\cdot 7e{-}007}\right) \\ -V_{cell}\cdot(3.5e{+}009\cdot[NO.]\cdot[O])$$

$$\frac{d([NO2.]\cdot V_{cell})}{dt} = +V_{cell}\cdot(3.5e{+}009\cdot[NO.]\cdot[O]) \\ -V_{cell}\cdot(1.4e{+}010\cdot[NO2.]\cdot[.OH]) \\ +V_{cell}\cdot(100000\cdot[ONOOH]\cdot[H2O2]) \\ -V_{cell}\cdot(3.4e{+}007\cdot[NO2.]\cdot["O2.-"]) \\ -V_{cell}\cdot(1e{+}006\cdot[NO2.]\cdot[LH])$$

$$\frac{d([O]\cdot V_{cell})}{dt} = -V_{cell}\cdot(3.5e{+}009\cdot[NO.]\cdot[O])$$

$$\frac{d([ONOOH]\cdot V_{cell})}{dt} = +V_{cell}\cdot(1.4e{+}010\cdot[NO2.]\cdot[.OH]) \\ -V_{cell}\cdot(100000\cdot[ONOOH]\cdot[H2O2])$$

$$\frac{d(["H+"]\cdot V_{cell})}{dt} = -V_{cell}\cdot(500000\cdot["O2.-"]\cdot["O2.-"]\cdot["H+"]) \\ +V_{cell}\cdot(100000\cdot[ONOOH]\cdot[H2O2]) \\ +V_{cell}\cdot(1e{+}006\cdot[NO2.]\cdot[LH])$$

$$\frac{d(["ONOO-"]\cdot V_{cell})}{dt} = +V_{cell}\cdot(3.4e{+}007\cdot[NO2.]\cdot["O2.-"]) \\ -V_{cell}\cdot(900000\cdot[TDPxr]\cdot["ONOO-"]) \\ -V_{cell}\cdot(3500\cdot[TXNr]\cdot["ONOO-"]) \\ -V_{cell}\cdot(7200\cdot[T[SH]2]\cdot["ONOO-"])$$

$$\frac{d([TDPxo]\cdot V_{cell})}{dt} = -V_{cell}\cdot(220000\cdot[TXNr]\cdot[TDPxo]) \\ +V_{cell}\cdot(900000\cdot[TDPxr]\cdot["ONOO-"])$$

$$\frac{d([TDPxr]\cdot V_{cell})}{dt} = +V_{cell}\cdot(220000\cdot[TXNr]\cdot[TDPxo]) \\ -V_{cell}\cdot(900000\cdot[TDPxr]\cdot["ONOO-"])$$

$$\frac{d\left(["NO2-"] \cdot V_{cell}\right)}{dt} = +V_{cell} \cdot (900000 \cdot [TDPxr] \cdot ["ONOO-"])$$

$$+V_{cell} \cdot (3500 \cdot [TXNr] \cdot ["ONOO-"])$$

$$+V_{cell} \cdot (7200 \cdot [T[SH]2] \cdot ["ONOO-"])$$

$$+V_{cell} \cdot (1e+006 \cdot [NO2.] \cdot [LH])$$

$$\frac{d\left(["MGO.-"] \cdot V_{cell}\right)}{dt} = +V_{cell} \cdot (0.0085 \cdot [Arg] \cdot [MGO])$$

$$+V_{cell} \cdot (0.0068 \cdot [Lys] \cdot [MGO])$$

$$-V_{cell} \cdot (1.31e-010 \cdot ["MGO.-"] \cdot [O2])$$

Supplementary Information_2

Multiscale Process Modelling in Translational Systems Biology of *Leishmania major*: A Holistic view

Nutan Chauhan[1], Shailza Singh[1#]

[1]National Centre for Cell Science, NCCS Complex, Ganeshkhind, SP Pune University Campus, Pune-411007, India

[#]Corresponding author

Email:shailza_iitd@yahoo.com, singhs@nccs.res.in

Phone: +91-20-25708296/95

Fax: +91-20-25692259

Supplementary Figures:

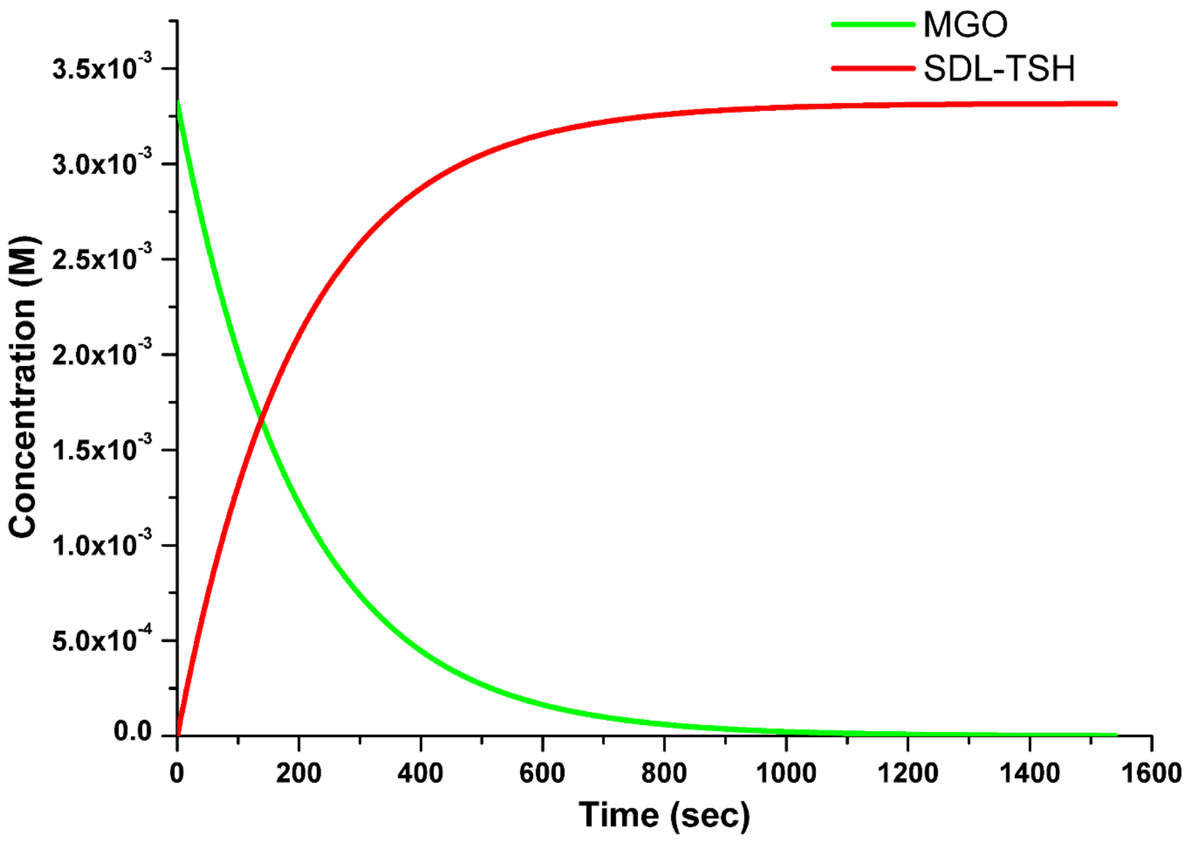

Figure S1. Prediction of MGO consumption and SDLTSH synthesis through time course simulation in basal kinetic model

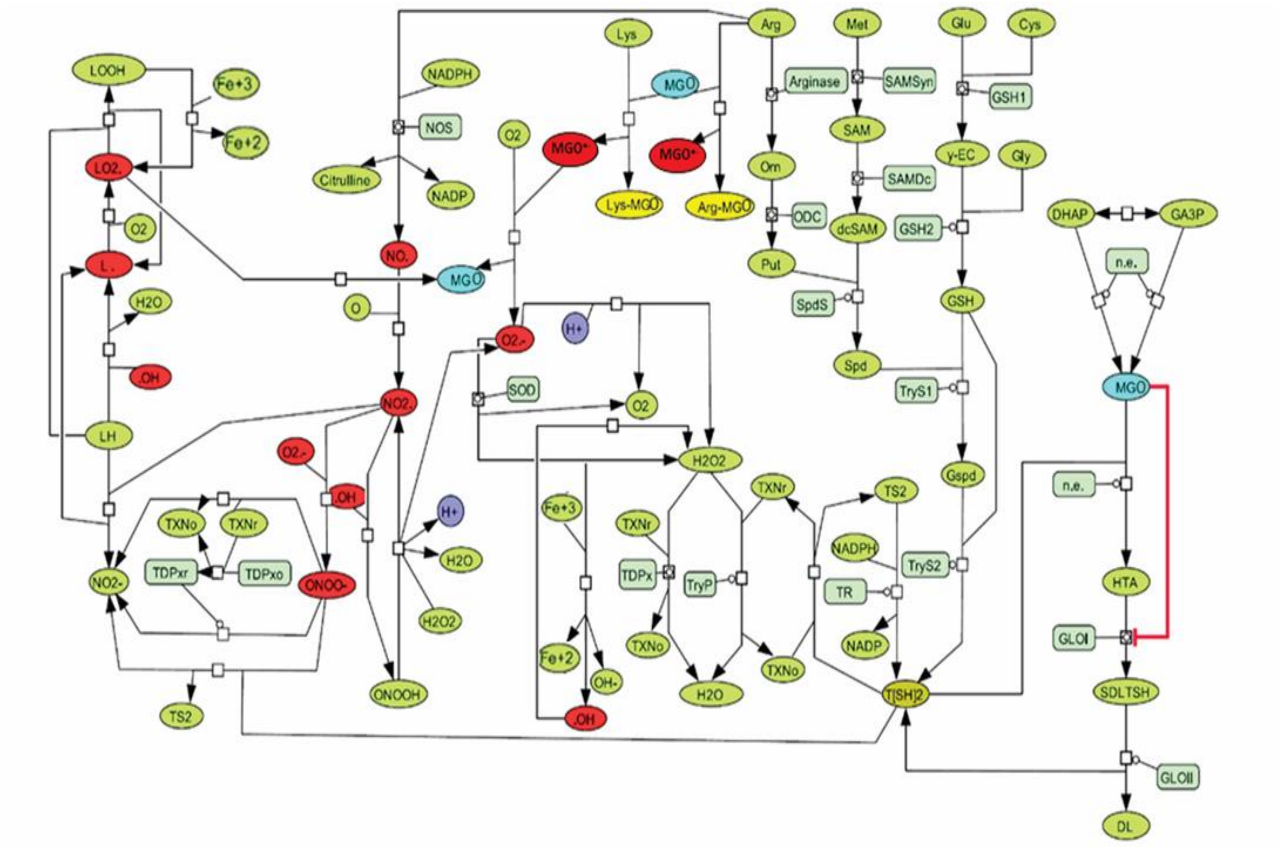

Figure S2. Illustration of large scale kinetic model used for prediction of free radicals and AGEs formation.

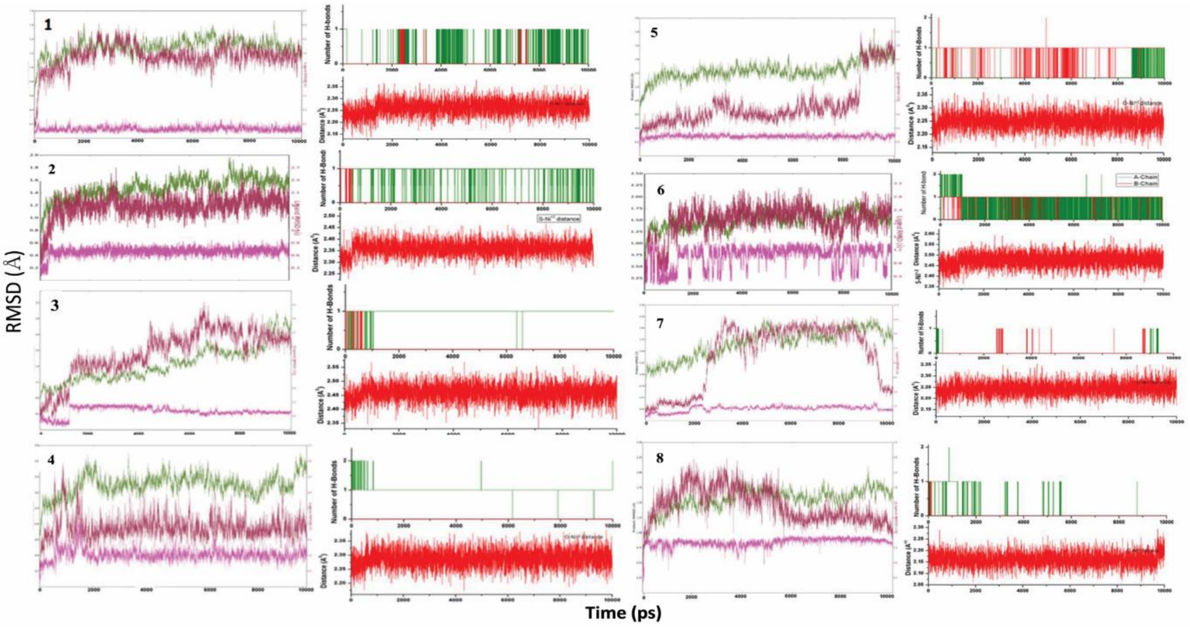

Figure S3. Evaluation of time course simulation of selected top eight ligand-GLO I complexes for 10 ns.

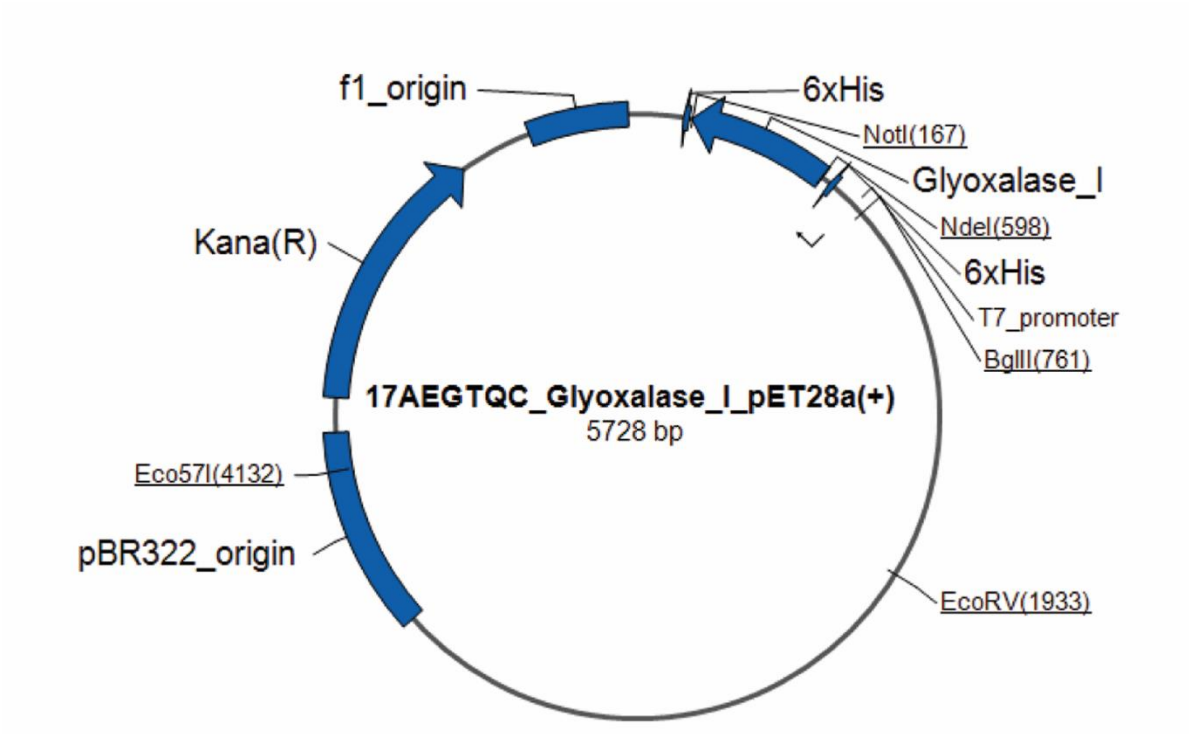

Figure S4. Glyoxalase I construct map procured from Invitrogen.

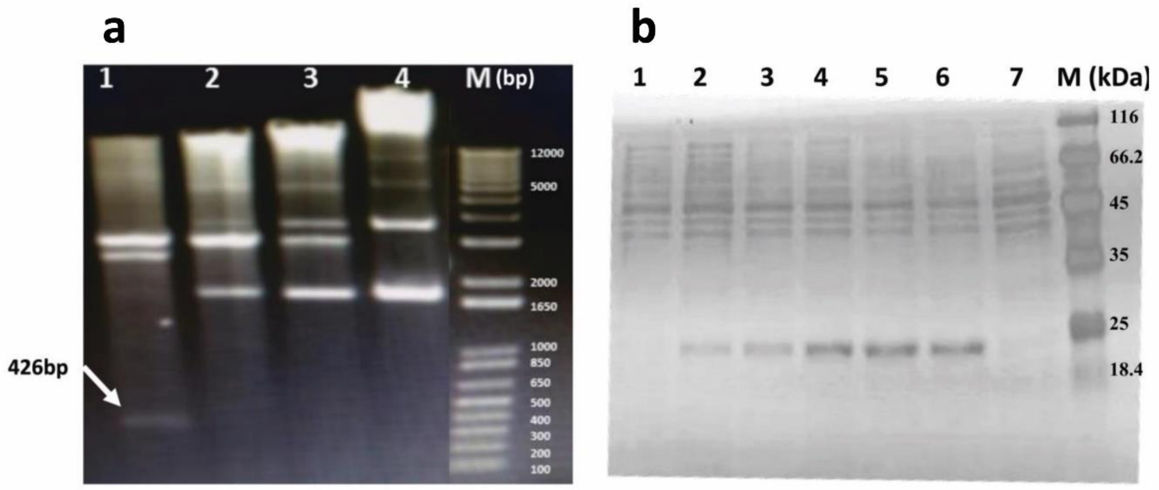

Figure S5. Gel electrophoresis of *gloI* gene and GLO I protein. **(a) I**ndicating the location of *gloI* gene. Lane 1: NdeI+NotI; 2: NdeI; 3: NotI; 4: Construct with GLOI; 5: DNA molecular weight marker. **(b)** SDS-PAGE indicating the location of GLOI protein. Lane 1: Noninduced 5 hr; 2: Induced 1hr; 3: Induced 2hr; 4: Induced 3hr; 5: Induced 4hr; 6: Induced 5hr; 7: Untransformed BL21 strain 5hr; M: Protein molecular weight marker.

Supplementary Tables:

Table S1. Kinetic parameters and initial concentration of metabolites used in the kinetic model system.

Reaction/ Enzyme name	Initial concentration (M)	k1 (s-1)	k-1 (s-1)	k2 (M-1.s-1)	Km (M)	V (M.s-1.mg-1)	s0.5 (M)	n (Hill coefficient)	Kd (M)	Kcat
TXN reduction				2.00E+02						
TDPx	28E-6 (TXNr)				2.2E-6 (txn) 193E-6 (H2O2)					15.4
TryP	28E-6 (TXNr)				4.9+-0.6 (TXN) 6.3+-0.8 (H2O2)					8.8
Fenton Reaction	8.8E-9 (Fe+2)			7.60E+01						
O2.- formation from MGO.-	1E-9 (O)			1.31E-10						
O2.- dismutation				5x10E+5						
SOD				1.6-1.8x10E+9						
NOS					4.9E-6 (Arg) 0.7E-6 (NADPH)	3.50E-06				
NO2. formation	1E-9 (O)			3.5x10E+9						
ONOOH formation				1.4x10E+10						
NO2. formation				1x10E+5						
ONOO- formation				3.4x10E+7 to 7x10E+9						
TDPx reduction	2E-7 (TDPx)			2.2x10E+5						
TDPx for NO2-				9x10E+5						
TXN for NO2-				3.5x10E+3						
T[SH]2 for NO2-				7.2x10E+3						
Lipid radical	0.025 (LH)			1.00E+10						
Lipid peroxide radical	0.00E+00			3x10E+8						
LOOH formation				5.00E+01						

Lipid radical from NO2.				1.00E+06						
Fenton Reaction				1.00E+04						
MGO synthesis				10E+6-7						
GSH1 (y-ECS)	1e-05 (Cys) 1e-05 (Glu)				9.2E-3 (Glu) 1.7E-3 (Cys)	1.80E-06			9.37E-05	
GSH2 (GS)	1e-05 (Gly)				4E-5 (yEC) 1.2E-3 (Gly)	3.40E-08			8.00E-05	
TR	5e-05 (TS2 and NADPH)				50E-6 (TS2) 20E-6 (NADPH)	5E-5 (TS2) 2E-5 (NADPH)				
Arginase					0.0215	2.40E-03				
ODC					4.20E-04	2.53E-02 (calculated)				7.7 +- 0.2
SAMsyn (MAT)	1.00E-05					3.40E-09	2.50E-04	2.3		0.32
SAMdc (AdoMetDC)					0.00038	5.20E-09				0.0013+-0.0004
SpdS					205E-6 (Put) 0.09E-6 (dcSAM)	1.98E-13				
TryS1					940E-6 (Spd) 89E-6 (GSH) 40E-6 (Gspd) 89E-6 (GSH)	8E-07 (calculated)				2
TryS2										2
MGO to HTA	9.00E-01			5.60E-03						
HTA to MGO		0.016								
(GLOI)					3.20E-05	1.59E-4 (calculated)				800
(GLOII)					3.90E-05	2.30E-09				
Arginine modification	2.00E-03		4.40E-06	8.50E-03						
Lysine modification	2.00E-03		5.00E-04	6.80E-03						

Table S2. Robustness of the basal kinetic model (Model 1).

Vmax GLO I	Folds	SDLTSH
$1.592x10^3$	0.1x	0.0033
$1.592x10^4$	1x	0.0033
$1.592x10^5$	10x	0.0033
**Km GLO I**		
$3.2x10^4$	0.1x	0.0033
$3.2x10^5$	1x	0.0033
$3.2x10^6$	10x	0.0033

Table S3. Details of selected top 8 compounds after ADME/Tox and docking

Comp no.	Docking Score	MW#	RO5*	PAINS Filter$
C1	-7.746	232.34	0	Pass
C2	-7.15	259.35	0	Pass
C3	-8.002	301.36	0	Pass
C4	-7.595	238.28	0	Pass
C5	-7.592	293.32	0	Pass
C6	-6.694	247.35	0	Pass
C7	-6.588	246.30	0	Pass
C8	-6.587	227.22	0	Pass

#**MW:** molecular weight; * **RO5:** Lipinski's Rule of five to evaluate druglikeness (No more than 5 hydrogen bond donor, No more than 10 hydrogen bond acceptors, A molecular mass less than 500 daltons, An octanol-water partition coefficient (log *P*) that does not exceed 5). "0" means the compound passed the RO5; $**PAINS filters:** Pan-assay interference compounds. PAINS are chemical compounds that often give false positive results in high-throughput screens. They represent poor choices for drug development and yet can furnish data that in isolation may be suggestive of a selective and optimizable hit.

Table S4. Top 10 compounds selected on the basis of their docking score after pharmacophore based screening and molecular docking

	ZINC id	Docking score	MW
1	ZINC72199852	-11.229	311.519
2	ZINC06188149	-10.577	337.224
3	ZINC72199240	-10.537	255.624
4	ZINC04749669	-10.307	341.363
5	ZINC71689273	-10.059	279.378
6	ZINC13496706	-10.006	299.252
7	ZINC13496705	-9.887	303.671
8	ZINC71688725	-9.804	265.352
9	ZINC18066480	-9.675	303.671
10	ZINC71690425	-9.579	255.288

Supplementary File 3

Multiscale Process Modelling in Translational Systems Biology of *Leishmania major*: A Holistic view

Nutan Chauhan[1], Shailza Singh[1#]

[1]National Centre for Cell Science, NCCS Complex, Ganeshkhind, SP Pune University Campus, Pune-411007, India

[#]Corresponding author

Email:shailza_iitd@yahoo.com, singhs@nccs.res.in

Phone: +91-20-25708296/95

Fax: +91-20-25692259

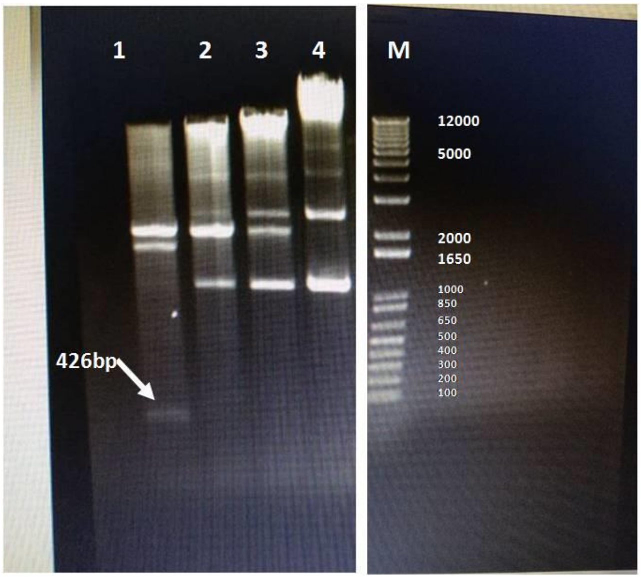

Figure Sa: Gel electrophoresis of *gloI* gene and GLO I protein. **(a) I**ndicating the location of *gloI* gene. Lane 1: NdeI+NotI; 2: NdeI; 3: NotI; 4: Construct with GLOI; 5: DNA molecular weight marker.

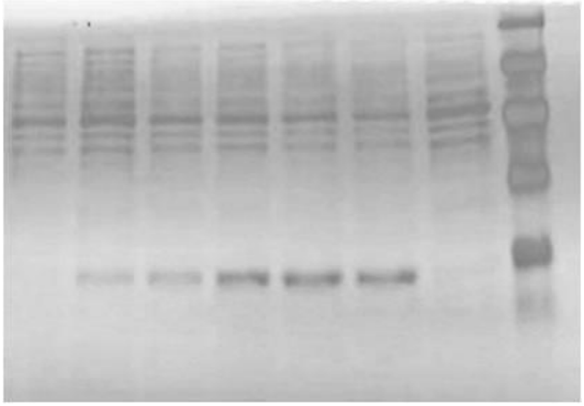

Figure Sb: SDS-PAGE indicating the location of GLOI protein. Lane 1: Noninduced 5 hr; 2: Induced 1hr; 3: Induced 2hr; 4: Induced 3hr; 5: Induced 4hr; 6: Induced 5hr; 7: Untransformed BL21 strain 5hr; M: Protein molecular weight marker.

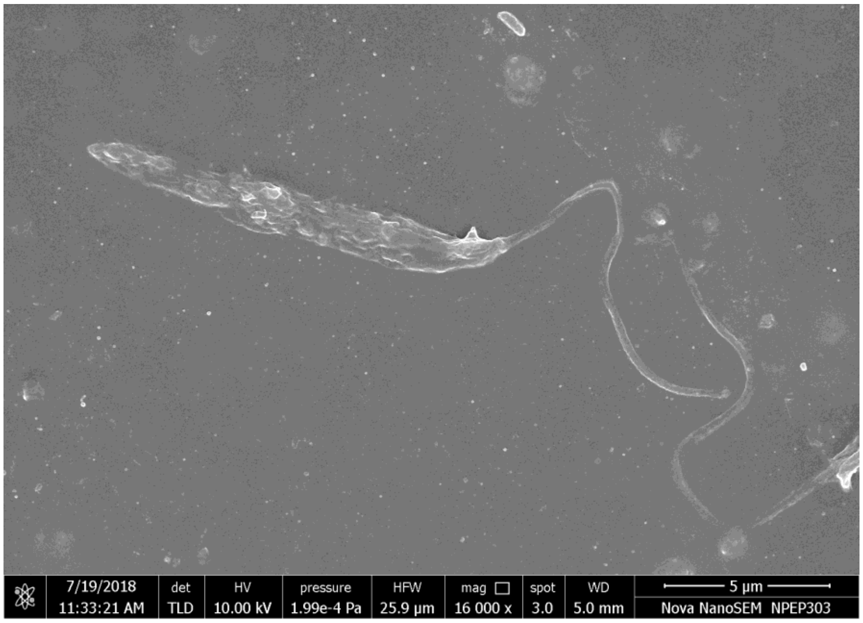

Figure 9a. Morphological analysis of untreated Leishmanial promastigotes through SEM.

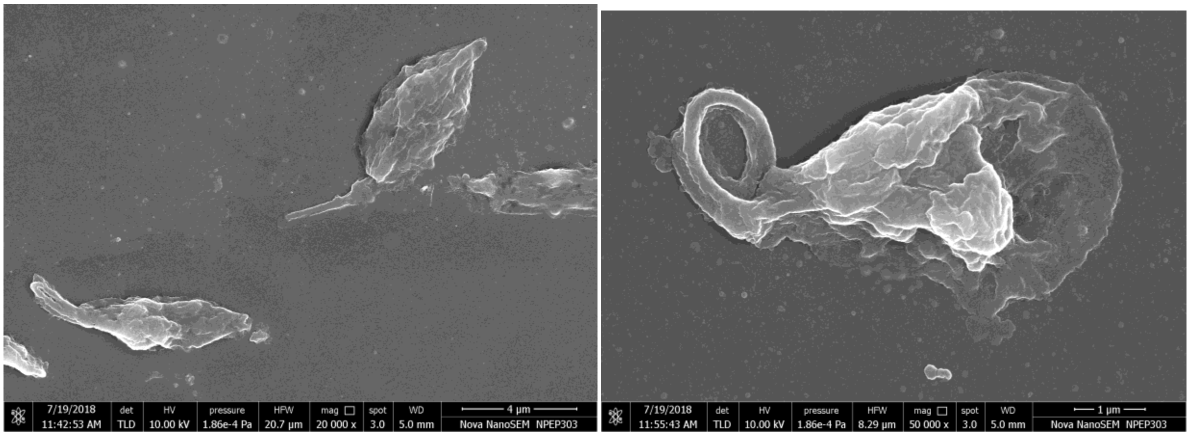

Figure 9bc. Morphological analysis of C1 treated Leishmanial promastigotes through SEM.

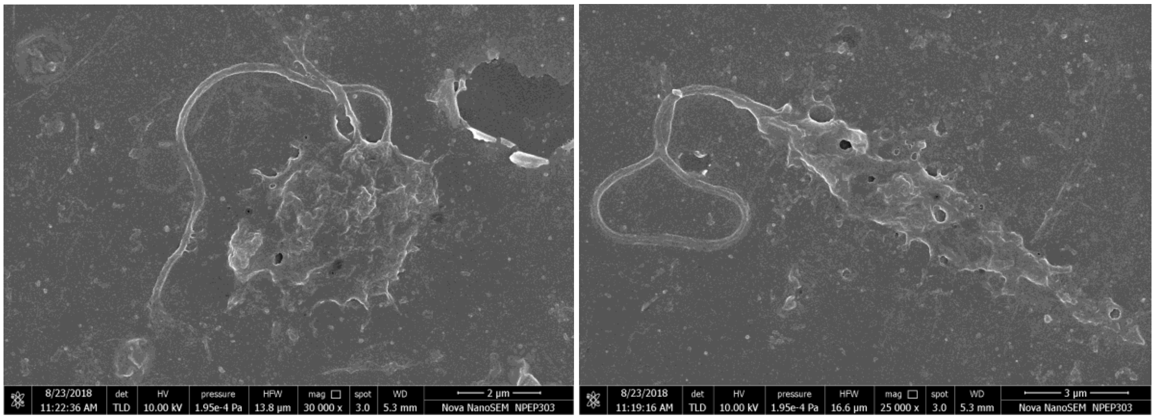

Figure 9de. Morphological analysis of C2 treated Leishmanial promastigotes through SEM.
